# Analysis of immunogenicity and purification methods in conjugated polysaccharide vaccines: a new approach in fighting pathogenic bacteria

**DOI:** 10.3389/fimmu.2024.1483740

**Published:** 2024-11-20

**Authors:** Arya Sheikhi, Mina Shirmohammadpour, Nima Mahdei Nasirmahalleh, Bahman Mirzaei

**Affiliations:** ^1^ Department of Microbiology and Virology, Zanjan University of Medical Sciences, Zanjan, Iran; ^2^ Student Research Committee, Department of Medical Microbiology and Virology, School of Medicine, Zanjan University of Medical Sciences, Zanjan, Iran; ^3^ Department of Medical Biochemistry, School of Medicine, Zanjan University of Medical Sciences, Zanjan, Iran

**Keywords:** glycoconjugate, vaccine, polysaccharide, conjugate, polysaccharide vaccine, immunogenicity, conjugate vaccine, antibacterial vaccine

## Abstract

Carbohydrates are commonly found in conjunction with lipids or proteins, resulting in the formation of glycoconjugates such as glycoproteins, glycolipids, and proteoglycans. These glycoconjugates are essential in various biological activities, including inflammation, cell-cell recognition, bacterial infections, and immune response. Nonetheless, the isolation of naturally occurring glycoconjugates presents challenges due to their typically heterogeneous nature, resulting in variations between batches in structure and function, impeding a comprehensive understanding of their mechanisms of action. Consequently, there is a strong need for the efficient synthesis of artificial glycoconjugates with precisely described compositions and consistent biological properties. The chemical and enzymatic approaches discussed in this paper present numerous research opportunities to develop customised glycoconjugate vaccines.

## 1 Introduction

Glycoconjugation in biochemistry involves attaching carbohydrate molecules to proteins, lipids, or other biomolecules. Over the past two decades, the study of carbohydrates and glycoconjugates has gained significant attention in biochemistry and cell biology, as research has shown they play essential roles in various biological processes (1–4). Progress in glycoconjugate research has led to the development of glycoconjugate vaccines, showing promise in clinical trials (5–9). Conjugate vaccines, created by attaching bacterial polysaccharides (PSs) to carrier proteins, have proven to address the shortcomings of unconjugated PS vaccines. At the same time, the latter is effective in adults; these vaccines typically do not generate immunological memory or sustained antibody production in children under 2 years, and their antibody affinity maturation is inferior to that of conjugate vaccines. Additionally, repeated administration of the same PS antigen may lead to hypo-responsiveness. Conjugating bacterial PSs to carrier proteins elicits an immunogenic response that shifts from T-cell-independent to T-cell-dependent mechanisms. Specifically, a reduced PS chain of defined molecular weight is activated through periodate oxidation or cyanylation to facilitate interaction with a carrier protein that possesses T-cell epitopes. T-cell responses in infants manifest significantly earlier than B-cell responses, with T-cells exhibiting adult-like immunophenotype by 5 months. The T-cell assistance from glycoconjugate protein epitopes enables carbohydrates, typically T-cell-independent antigens, to elicit durable and boostable IgG antibody responses in children under 2 years. Ultimately, glycoconjugate vaccines demonstrate superior efficacy in adults compared to PS vaccines and have a greater capacity to reduce *meningococcal* carriage and transmission, a benefit not consistently observed with plain PS vaccines (10, 11). Further research is needed to clarify these mechanisms and to develop more efficient synthesis methods (1).

This review offers a detailed analysis of biochemical techniques for producing glycoconjugate vaccines.

## 2 Techniques for purification of bacterial vaccines

Purifying PSs is essential in bacterial vaccine production to ensure safety and efficacy. Researchers have developed and refined various methods to purify these PSs efficiently.

### 2.1 The purification process of capsular polysaccharide produced by *Haemophilus influenzae* type b

Although there have been numerous investigations into the immunogenic properties of PS vaccines, the methods for producing and purifying them on a large scale are not widely known, and the available literature on the subject is limited, consisting mainly of patents. The virulence of *Haemophilus influenzae* (*H. influenzae*) type b (Hib) is primarily due to its capsular polysaccharide (CPS), a repeating polymer of ribosyl ribitol phosphate (PRP). The process of purifying PRP commences with the inactivation of cellular components utilising agents such as phenol, formaldehyde, or thimerosal, followed by the isolation of the culture medium through centrifugation. Subsequently, the clarified supernatant is concentrated via tangential flow ultrafiltration using a membrane with a molecular weight cut-off of 50 kDa, after which the polysaccharides are precipitated with the cationic detergent cetyltrimethylammonium bromide (cetavlon), after which the resulting PRP-cetavlon complex is solubilised with calcium chloride, accompanied by multiple rounds of ethanol precipitation and desproteinization through phenol extraction and additional ethanol precipitation. Ultracentrifugation techniques are used to separate lipopolysaccharides (LPS) from the PRP (12).

Researchers in the laboratory have developed a new technique for purifying vaccines against *Neisseria meningitidis* (*N. meningitidis*) serotype C and *Streptococcus pneumoniae* (*S. pneumoniae*) serotypes 23 and 6B, the process of phenol precipitation, employed for desproteinization, and the technique of ultracentrifugation, utilised for the elimination of LPS, have been supplanted by enzymatic treatment and ultrafiltration in conjunction with a chelating agent and detergent. The newly implemented methodologies offer the advantage of omitting toxic and corrosive solvents like phenol while reducing the volume and frequency of ethanol precipitation—a reagent known for its explosive potential. Furthermore, ultracentrifuges are expensive, and the process’s operational capacity is limited by the volume each centrifuge can accommodate. Despite the potential for enzymatic treatment and tangential ultrafiltration to augment downstream processing expenses, the novel purification approach described herein stands out for its simplicity, efficiency, non-toxicity, scalability, and environmental sustainability (12). This method was later improved upon; the culture broth underwent centrifugation for cell separation, followed by modified tangential flow ultrafiltration as per Takagi et al. (13). A polyethersulfone spiral membrane with a 100 kDa nominal molecular weight cutoff was utilised for the washing of the concentrated sample using six different buffer volumes. The concentrated fraction, termed first concentration by tangential ultrafiltration—1CTUF100, was precipitated with ethanol, yielding soluble and insoluble fractions. The PS was extracted from the insoluble fraction using deionised water, with subsequent centrifugation yielding a water-soluble fraction. The pH of the water-soluble PS was adjusted and subjected to enzymatic treatments involving endonuclease and various proteases under specified conditions. The enzymatic hydrolysis was followed by diafiltration using the same conditions as the initial concentration, producing the Purified PSb—Enz+ 2CTUF100 fraction. Previous studies indicated a 68% yield of purified PS; however, the purity of PSb relative to protein was inadequate. Additional washing with detergents and chelating agents was incorporated into the initial and final purification steps to enhance purity (14).

Centrifugation techniques are commonly utilised within the pharmaceutical industry to segregate bacteria from the culture broth. Nevertheless, tangential microfiltration systems provide a cost-efficient substitute for centrifugation, rendering it an appealing choice for reducing production expenditures. Specifically, in manufacturing the Hib vaccine, the substitution of centrifugation with tangential microfiltration has the potential to lower expenses and enhance the availability of the vaccine in economically disadvantaged areas. Centrifugation incurs more significant capital costs relative to filtration. Therefore, employing a reusable tangential microfiltration system may enhance cost-effectiveness in Hib vaccine production. In PRP isolation, Hib culture broth undergoes centrifugation to eliminate bacterial cells. The PS, approximately 622 kDa, is purified from the supernatant through tangential ultrafiltration after ethanol precipitation. The selection of microfiltration systems for Hib cell separation is influenced by membrane material properties, device architecture, and process parameters, including pressure and temperature. For clarity, these factors are categorised as equipment and process parameters. First, the membrane material’s composition significantly affects recovery rates, with distinct outcomes observed for Durapore, hollow fibre, and Hydrosart membranes. Second, membrane architecture influences fouling prevention and enhances turbulence and shear rate during the culture broth’s flow. For instance, the Prostak membrane exhibited lower recovery than the Pellicon membranes despite identical material. Third, the membrane area relative to culture broth volume is critical to prevent overloading. Process duration and feasibility are also contingent on transmembrane flow, which correlates with membrane area. Process parameters are primarily regulated by retentate pressure and feed flow. Small-scale trials revealed that increased transmembrane pressure and feed flow enhance filtrate flux. It was also noted that PRP retention in membranes decreases linearly with rising TMP, while feed flux is similarly influenced. The scaling of the process from 50 cm² to 1 m² and 0.3 L to 7 L of broth demonstrated a linear relationship. Pilot-scale separation achieved approximately 87% PRP recovery, while lab-scale experiments neared 100%. In summary, tangential filtration is a compact, eco-friendly method showing promising outcomes for cell separation and PRP recovery from Hib. While further optimisation of the pilot scale process is necessary, tangential microfiltration presents a viable alternative to centrifugation (15).

### 2.2 The purification process of capsular polysaccharides of *Streptococcus pneumoniae*

The purification of capsular polysaccharides (CPS) is essential in developing vaccines against *S. pneumoniae*, whether composed of CPS alone or CPS linked to proteins. Although these vaccines are effective and safe, they are costly. There have been endeavours to enhance the efficiency and output of CPS purification methods and to streamline the process. Numerous publications have addressed the purification of *S. pneumoniae* CPS. These works typically present enhancements to initial procedures that improve purification outcomes. Following fermentation, it is common practice to isolate cell cultures via centrifugation and lyse them with sodium deoxycholate. Alternatively, some researchers introduce sodium deoxycholate post-fermentation but pre-cell collection, extracting CPS from the resulting supernatant or filtrate. A preferred method involves bacterial inactivation with thimerosal and subsequent cell removal via filtration, as detergent lysis can compromise cell membranes, releasing intracellular contaminants that complicate purification. The primary contaminants necessitating removal are proteins, nucleic acids, and cell-wall carbohydrates. The World Health Organization (WHO) stipulates that acceptable protein and nucleic acid contaminant levels are below 3% and 2%, respectively. Classical processes emphasise the necessity of contaminant removal and the drawbacks of specific toxic reagents, which introduce significant intracellular contaminants. Consequently, incorporating additional purification steps complicates the process, reducing yields and increasing costs. Innovative capsule purification methods have been evaluated to enhance yield and purity. Despite removing or substituting some laborious steps, a multistep approach remains prevalent. Recently developed purification techniques seek to improve CPS purity and yield with a more streamlined workflow. Most methods are tailored to specific CPS types but can potentially be modified for others with analogous physicochemical characteristics (16–20). While continuous culturing and chemically defined media can aid in simplification. Adjusting the composition of growth media and culture conditions to optimise CPS production is a crucial research focus (17).

Experimental investigations were conducted to assess the impact of membrane pore dimensions, filtrate flux, and solution parameters on the transmittance of both the conjugate and the unbound PS across various ultrafiltration membranes. The purification of the conjugate was executed utilising diafiltration carried out within a linearly scalable tangential flow filtration (TFF) cassette. A removal efficiency exceeding 98% of the unbound PS was achieved within a five-diavolume diafiltration procedure, representing a notable advancement compared to previously documented outcomes for the purification of analogous conjugated vaccines. These findings unequivocally illustrate the potential for ultimately employing ultrafiltration and diafiltration techniques to purify conjugated vaccine formulations (21).

A novel methodology is engineered for universal applicability across all 15 serotypes in an imminent 15-valent conjugate vaccine. A decision-making step has been integrated to ascertain the utilisation of supernatant or precipitate based on the charge of the target CPS post-CTAB precipitation. This technique amalgamates various methods, including acid and ethanol precipitation alongside HA chromatography, to attain elevated CPS purity and minimise cell wall polysaccharide (CWPS) contamination. This approach notably prioritises CWPS reduction, a critical contaminant in CPS purification, diverging from conventional studies that mainly focus on nucleic acid and protein impurities. While traditional multi-step methods tend to diminish CPS yield, this strategy markedly lessens CWPS contamination. A comparable method utilised in the production of Prevenar 13, a reference vaccine in the immunogenicity assessment, also employs CTAB precipitation; however, it differentiates by utilising NaI for CTAB precipitation, whereas this method adopts ethanol precipitation for CPS. In summary, this CPS purification methodology integrates established separation techniques, such as ultrafiltration, CTAB precipitation, and chromatography, yet confronts challenges related to CWPS contamination. Process optimisation was conducted to mitigate these issues, significantly reducing CWPS levels, which subsequently improved immunogenicity when the CPS was incorporated into a conjugate vaccine. Refinement of the primary purification processes, notably extensive ultrafiltration/diafiltration and impurity precipitation, resulted in a substantial decrease in CWPS concentrations. ELISA and OPA evaluations in animal models further indicated that enhanced CPS quality positively influenced the immunogenicity of the multivalent *pneumococcal* conjugate vaccine. These results underscore that the diminished CWPS contamination, attained through this optimised methodology, significantly elevates CPS quality and facilitates the advancement of innovative multivalent conjugate vaccines against *pneumococcal* diseases (22).

A new study investigated a novel aeration and sterilisation approach using formaldehyde or β-propiolactone (BPL) to increase soluble PS yield and inhibit bacterial lysis. Furthermore, a refined CPS purification protocol was established, utilising ultrafiltration, diafiltration, acid and alcohol precipitation, and concluding with diafiltration and lyophilisation for pure PS production. CPSs prepared via formaldehyde and BPL exhibited markedly lower residual impurities than those produced through conventional deoxycholate sterilisation. This innovative CPS preparation methodology was also shown to be scalable for PS vaccine manufacturing (23).

Efficiencies in the purification processes for *S. pneumoniae*, *H. influenzae type b*, and *N. meningitidis* have been achieved by minimising ethanol precipitations and discontinuing phenol. Various modifications and techniques, including protease digestion, diafiltration, O-acetylation, hydrophobic interaction chromatography, and enzymatic hydrolysis, have been suggested for *meningococcal* and Hib CPS, which could be considered for *pneumococcal* CPS purification. The choice of downstream procedures, such as ethanol precipitation and refining steps, depends on serotype variations and CPS characteristics. Nonetheless, further research is required in the realm of *pneumococcal* vaccine production (17).

### 2.3 The purification process of capsular polysaccharides of *Neisseria meningitidis*

The virulence of various *N. meningitidis* serogroups is attributed to their distinct PS capsules. Among the 13 identified serogroups of *N. meningitidis*, serogroups A and C represent the most predominant strains associated with *meningococcal* diseases that are preventable through conjugate vaccination. Consequently, a necessity exists for developing cost-effective multivalent *meningococcal* conjugate vaccines, ideally incorporating conjugates for serogroups A and C, which can be accessible to populations in low-income countries. The advancement in the synthesis of PS conjugate vaccines represents a significant leap forward in preventing bacterial infections, exemplified by *meningococcal* disease. Nevertheless, developing nations encounter considerable obstacles in producing these vaccines, primarily attributable to the high costs and intricate nature of the purification methodologies necessitated to achieve the stringent purity levels required for the efficacy and safety of vaccines. The absence of accessible and streamlined techniques for purifying bacterial PSs has resulted in a reliance on well-resourced and established laboratories in developed countries. Moreover, the purification process for these PSs entails the utilisation of expensive reagents, labour-intensive procedures, and often prolonged processing times, which cumulatively exacerbate the overall cost of conjugate vaccines. Researchers are concentrating on innovating expedited, economically viable, and scalable purification techniques for PS antigens to surmount these challenges. This investigation delineates optimised protocols for purifying CPSs derived from *N. meningitidis* serogroups A and C (MenA-PS and MenC-PS), which are essential to vaccines directed against these pathogens. By enhancing and expediting procedures such as CTAB-based precipitation and incorporating hydrophobic interaction chromatography (HIC) for MenC-PS, this methodology diminishes processing durations by more than 70% while attaining the purity benchmarks established by the WHO. The findings of this research indicate that these novel techniques are both amenable to industrial scaling and economically feasible, thereby providing a pragmatic approach to mitigating the cost and intricacy of vaccine production in resource-constrained environments, ultimately facilitating broader access to life-saving conjugate vaccines (24).

## 3 Understanding conjugation in the context of bacterial vaccines

**Figure 1:**
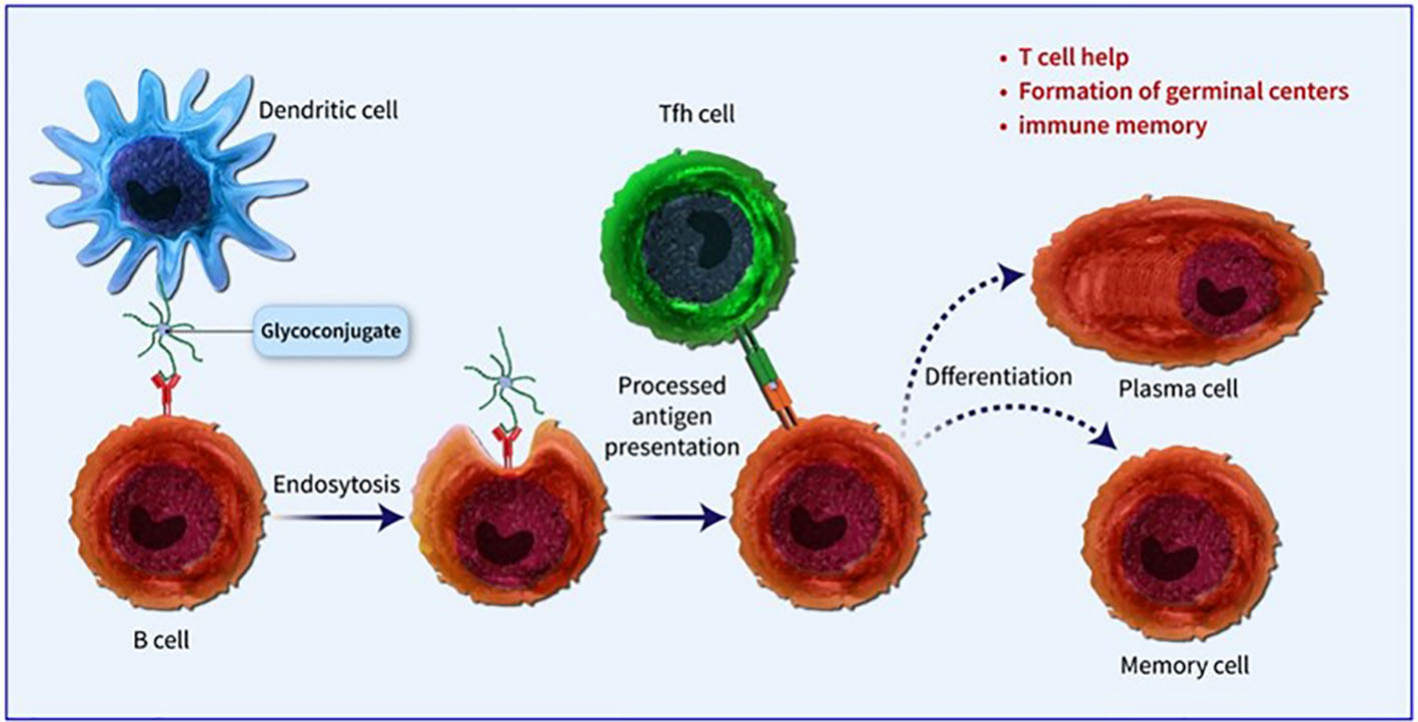
Dendritic cells located at the immunization site absorb the conjugate vaccine, which is then carried to lymph nodes and the spleen, initiating the development of germinal centres. Within these centres, B cells attach to the surface of dendritic cells and take in both the carbohydrate and protein elements of the vaccine. The protein element is processed and displayed to Tfh cells, leading to the stimulation and transformation of B cells into plasma cells and memory B cells.

**Figure 2:**
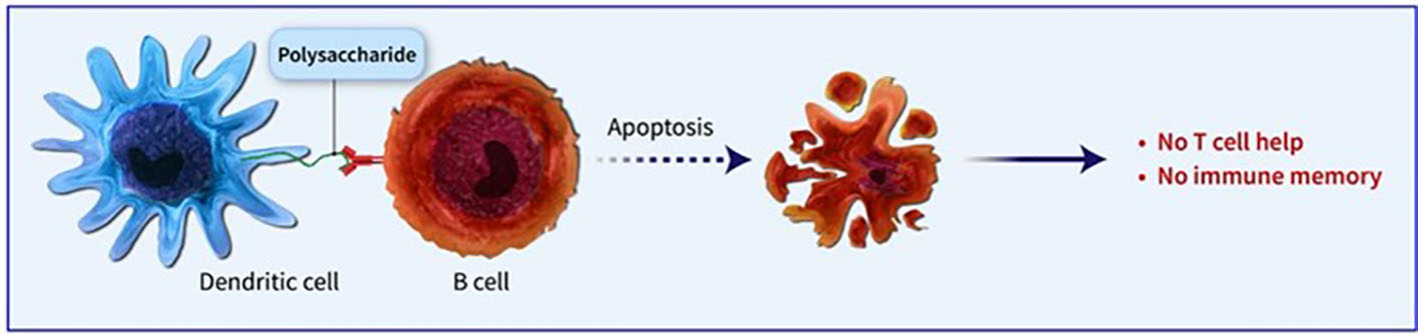
PS immunization may result in the death of memory B cells and diminish the response to future immunizations, a condition known as hyporesponsiveness.

Conjugated vaccines are comprised of a feeble antigen (hapten) that exclusively contains B-cell epitopes, typically a microbial PS or oligosaccharide (OS), which is covalently bonded to a protein serving as a source of T-cell epitopes (carrier). The concept of the hapten carrier originated in 1929 with Goebel and Avery (25). Investigations were carried out during the earlier frame to examine the significance of surface PSs in bacterial virulence and the protective function of antibodies against *pneumococcal* PS. These studies prompted the development of the initial PS-based vaccine aimed at specific *pneumococcal* serotypes, prepared for implementation in 1945 (26). John Robbins’ team later utilised the research conducted by Goebel and Avery in the 1930s in the 1970s to develop the first glycoconjugate vaccine using the Hib PS (27). In the latter part of the 1970s, a quadrivalent PS vaccine aimed at *N. meningitidis* was approved for use, succeeded by a 23-valent anti-*pneumococcal* PS vaccine and, subsequently, the anti-Hib PS vaccine. Despite showing some efficacy in adults, these vaccines fell short of offering adequate protection to vulnerable populations, especially infants and children below two years of age (28). This prompted the exploration of glycoconjugate vaccines and the reintroduction of the concept of carrier/hapten, initially introduced by Goebel and Avery. The initial glycoconjugate vaccines targeting Hib received official approval from 1987 to 1990 (29). The initial synthetic glycoconjugate vaccine targeting Hib received approval for human usage in Cuba in 2004, demonstrating the possibility of utilizing intricate carbohydrate-derived vaccines and paving the way for creating comparable strategies for different human pathogens (30). The carbohydrate portion found in glycoconjugates offers B epitopes to interact with B cells, while the protein portion presents T epitopes to solicit T-cell assistance. Dendritic cells located at the immunization site absorb the conjugate vaccine, which is then carried to lymph nodes and the spleen, initiating the development of germinal centres. Within these centres, B cells attach to the surface of dendritic cells and take in both the carbohydrate and protein elements of the vaccine. The protein element is processed and displayed to Tfh cells, leading to the stimulation and transformation of B cells into plasma cells and memory B cells (**Figure 1**). The carrier protein and saccharide can be administered together without a covalent bond as long as their interaction is robust enough to be introduced to B cells and absorbed in the same B cell for T-cell interaction. Glycopeptides may also be introduced and recognized by T-cell clones specific to carbohydrates. despite the significant effect of glycoconjugate vaccines on reducing infant mortality and illness, there is still a limited comprehension of their behaviour in diverse populations and enhancements to immunization schedules can be implemented. The immune reaction to conjugate vaccines differs based on age. For infants and young children, two doses are required for the initial immunization to achieve satisfactory protection. Although antibody levels decrease over time, they generally remain higher than those in children who have not been immunized. Additional doses can be administered using a different carrier, indicating the existence of memory B cells. Conversely, teenagers and adults likely already possess pre-existing memory B cells due to past exposure to the pathogen or related agents. PS vaccines can also trigger an antibody response in these age groups, albeit slightly weaker than conjugate vaccines. The response peaks after the first dose and does not enhance with the following doses. evidence indicates that PS immunization may result in the death of memory B cells and diminish the response to future immunizations, a condition known as hyporesponsiveness (**Figure 2**). The presence of unbound antibodies and carrier protein antibodies may impede the immune response by competing for the same antigen or obstructing antigen absorption by cells. Using specific carrier proteins in glycoconjugate vaccines may further heighten the risk of immune disruption (31). The examination of conjugate vaccines elucidates that the intricacies of their structure, particularly the number of saccharide chains linked to carrier proteins, can substantially affect immunogenicity; for conventional conjugates, an average of approximately 17 ± 5 chains per tetanus toxoid molecule is customary, whereas bioconjugates may encounter difficulties in attaining comparable chain densities, which could hinder their capacity to effectively cross-link B-cell receptors (32). Moreover, parameters such as dimensions and surface characteristics, including zeta potential, may influence the internalization by antigen-presenting cells (APCs) and the overall immunogenic profile (33). In summation, the structural characteristics and antigen presentation of conjugate vaccines are paramount for stimulating vigorous immune responses (34). The immunogenic potential of conjugate vaccines, notably those incorporating aluminium-based adjuvants, is significantly modulated by the extent of antigen adsorption onto alum. Empirical investigations suggest that the adsorption efficacy of antigens may fluctuate according to their pH, point of zero charge (PZC) of alum, ionic strength and isoelectric point of the antigen, consequently influencing the consistency and stability of the vaccine formulation over time. Various formulation methodologies, including sequential or competitive adsorption, can result in discrepancies in antigen distribution on alum particles, which subsequently affects the immune response (35). Furthermore, chemical conjugation techniques have been demonstrated to augment immune responses by enhancing the presentation of antigens, although irregular decoration may present certain obstacles (33). A comprehensive understanding of these adsorption mechanisms and their implications on immunogenicity is essential for the advancement of more effective vaccines that optimize antigen presentation and amplify immune activation (32, 33).

## 4 Conjugation techniques for bacterial vaccines

### 4.1 Traditional and semisynthetic

**Figure 3:**
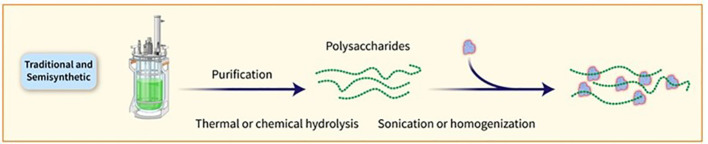
PSs can also be broken down through various techniques, such as thermal or chemical hydrolysis, sonication, or homogenisation. The resulting fractions are subsequently sized for more precise length and easier protein conjugation.

PSs are traditionally obtained through bacterial growth, harvesting, and purification steps. The conjugation of PSs to carrier proteins involves random linkage, bringing about high-molecular-weight and heterogeneous structures. Glycan-protein conjugation can be achieved through functional moieties or by derivatizing the PS with linkers. Chemical strategies such as NaIO4 oxidation and CDAP chemistry are commonly used for conjugation. Shorter OSs are preferred for manufacturing, as they facilitate conjugate production, filtration, and purification. The utilization of consistent and defined lower-molecular-weight PS groups can be accomplished by either chemical or mechanical splitting. Nevertheless, the isolation of PSs with specific lengths could impact the overall efficiency and intricacy of the procedure (11). Specific PS chemical groups may activate in certain cases before being attached to the carrier protein. One common method requires the interaction of hydroxy groups with cyanylating agents or carbonyldiimidazole to create active esters. As an alternative, amino groups can be incorporated using ammonium salts or dihydrazide spacers, which can then react with carboxylic acids of the protein or be linked to different bifunctional linkers for conjugation purposes. It is crucial to determine the optimal level of activation of the sugar chain to ensure effective conjugation without compromising the saccharide’s structural integrity. PSs can also be broken down through various techniques such as thermal or chemical hydrolysis, sonication, or homogenization, with resulting fractions subsequently sized for more precise length and easier protein conjugation (**Figure 3**). Chemical hydrolysis has proved to be capable of developing several commercially available glycoconjugate vaccines. Reduced-size PSs are typically linked to the protein using the end-reducing sugar, enabling selective modification and more well-defined structures. Numerous licensed glycoconjugate vaccines against specific pathogens are developed using these methodologies (36). Alternative methods for delivering PS antigens and carrier proteins in conjugate vaccines have been explored, such as the Protein Capsular Matrix Vaccine (PCMV) technology that uses crosslinked polymers to trap both components. The MAPS technology replaces covalent linkage with affinity-based coupling and has shown promise in a prototype *pneumococcal* vaccine, with further development underway for other pathogens (11).

### 4.2 Synthetic

**Figure 4:**
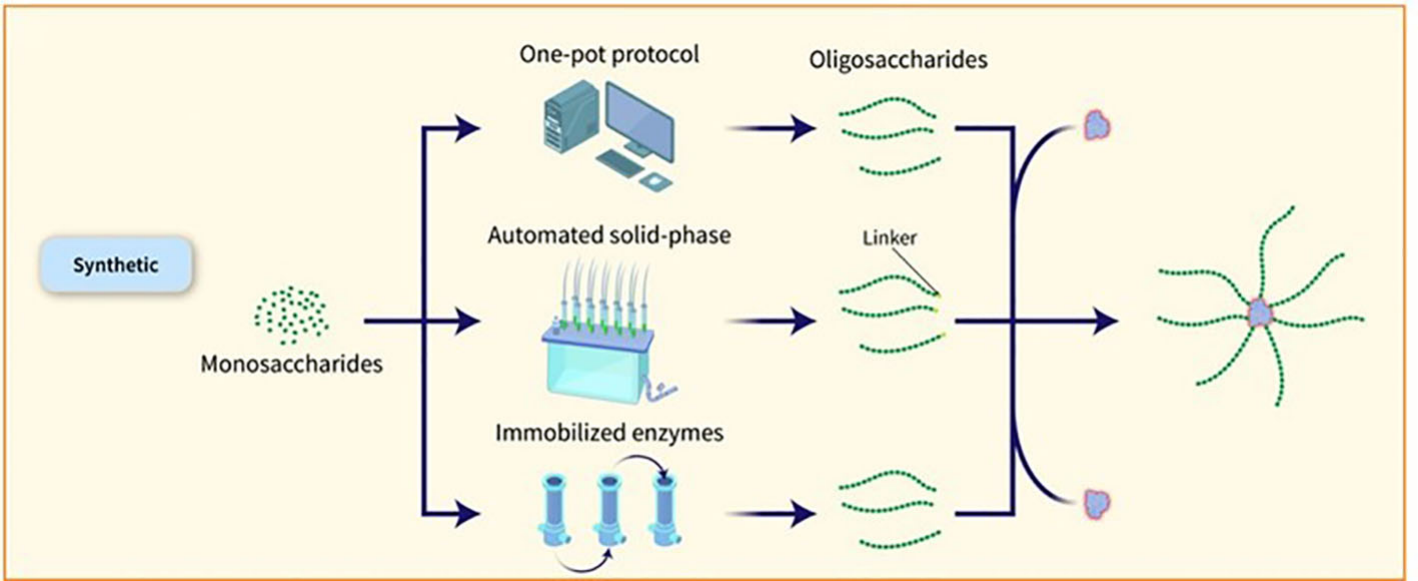
Various methods have been explored to expedite the assembly of glycans and obtain well-defined OS structures. One such method is the one-pot strategy, which allows for the amalgamation of multiple glycosylation steps into a single process, resulting in the production of target OSs in a shorter timeframe without the need for intermediate isolation or protective group manipulations. Databases containing reactivity values of building blocks have been developed to assist in the design of target OSs. Automated methods for synthesizing compounds, exemplified by the ‘Glyconeer’ synthesizer, have been created to facilitate the production of increasingly intricate and lengthy glycans. These automated systems have successfully synthesized diverse glycans, including complex structures and biologically relevant OSs. Additionally, glycan microarray and structural characterization methods have been used to identify crucial epitopes and select synthetic vaccine candidates. Synthetic glycoconjugate vaccines have been formulated using these methods, resulting in the production of antibodies with strong opsonophagocytic activity (11). The utilization of immobilized enzymes for a sequence of chemical and enzymatic processes has been applied to produce *N. meningitidis* serogroup X fragments.

The capacity to manufacture significant amounts of artificial carbohydrates for vaccine development has been proven in the case of the Quimi-Hib vaccine designed to combat Hib (30) and a synthetic glycoconjugate vaccine against *Shigella flexneri* (*S. flexneri*) serotype 2a has also been tested in clinical trials (37). Synthetic glycans present a promising strategy for advancing vaccines, particularly when acquiring natural PSs is difficult. Nevertheless, the extensive application of artificial sugars is impeded by elevated production expenses and intricate processes when contrasted with natural PSs. Nonetheless, recent progress in streamlining and hastening the creation of glycans and glycoconjugates using chemoenzymatic methods, one-pot procedures, and automated solid-phase synthesis is increasing the attractiveness of carbohydrate production for industrial purposes. By combining chemical or enzymatic synthesis techniques, targeted conjugation methods, and multi-component structures, it is possible to boost the immunological effects of glycoconjugates and streamline the development of more effective and safer vaccines by gaining a deeper insight into the factors influencing their effectiveness (38). Various methods have been explored to expedite the assembly of glycans and obtain well-defined OS structures. One such method is the one-pot strategy, which allows for the amalgamation of multiple glycosylation steps into a single process, resulting in the production of target OSs in a shorter timeframe without the need for intermediate isolation or protective group manipulations. Databases containing reactivity values of building blocks have been developed to assist in the design of target OSs. Automated methods for synthesizing compounds, exemplified by the ‘Glyconeer’ synthesizer, have been created to facilitate the production of increasingly intricate and lengthy glycans. These automated systems have successfully synthesized diverse glycans, including complex structures and biologically relevant OSs. Additionally, glycan microarray and structural characterization methods have been used to identify crucial epitopes and select synthetic vaccine candidates. Synthetic glycoconjugate vaccines have been formulated using these methods, resulting in the production of antibodies with strong opsonophagocytic activity (11). The utilization of immobilized enzymes for a sequence of chemical and enzymatic processes has been applied to produce *N. meningitidis* serogroup X fragments (**Figure 4**). This has resulted in creating an on-column technique that quickly produces conjugation-ready OSs of an ideal size for vaccine effectiveness. The process commences with a fully synthetic trisaccharide acceptor and a truncated immobilized form of the *N. meningitidis* X capsular polymerase (CsxA) (39).

### 4.3 Site-selective conjugation

**Figure 5:**
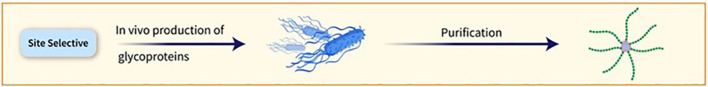
The conjugation of PSs to carrier proteins can occur randomly or at predetermined sites. Selective conjugation procedures can be achieved by targeting specific residues on the protein surface or using enzymatic chemistries.

The conjugation of PSs to carrier proteins can occur randomly or at predetermined sites. Selective conjugation procedures can be achieved by targeting specific residues on the protein surface or using enzymatic chemistries (**Figure 5**). Proteins incorporating unnatural amino acids can also be used for particular conjugation. Additionally, *in vivo* production of glycoproteins allows for the placement of the conjugation site at specific locations on the carrier protein (23). vaccines using this method have been tested in Phase-1/2 trials (27, 28).

## 5 Challenges and considerations in conjugation and purification

The challenges posed by conjugate vaccines are manifold and require careful consideration. One such challenge is the difficulty in purifying the PS component of these vaccines at high yields. This is due to the complex nature of PSs and the need for meticulous purification techniques to ensure their safety and efficacy. Additionally, the handling of pathogens during the production process presents another hurdle. Pathogens are inherently hazardous and require specialized facilities and protocols to guarantee the safety of both the workers and the final product. Moreover, there are multiple steps involved in the production of conjugate vaccines, each of which demands precise execution and monitoring. This not only adds to the complexity of the manufacturing process but also increases the time and cost involved. Furthermore, the analytical controls required for quality assurance are extensive and demanding. Rigorous testing and analysis are necessary to ensure the potency, purity, and consistency of the vaccines. Lastly, the cost of goods for conjugate vaccines is significantly higher compared to those derived from bacterial carbohydrates (31).

## 6 Successful applications of conjugation in bacterial vaccine development

In this table (**Table 1**) successful applications of conjugate vaccines have been categorized and expanded upon.

**Table 1:** Successful applications of conjugation in bacterial vaccine development.

Bacteria	Vaccines	Basis	Specific vaccine composition	Case study
Hib	Quimi-Hib	Hib conjugate vaccines are made by attaching Hib CPS to proteins, showing unique safety and structure (67). These vaccines effectively prevent Hib meningitis in countries with mass vaccination programs, significantly decreasing neonatal meningitis cases in developed nations (68).	The composition of the Quimi-Hib vaccine includes synthetic PRPs, which has an average of eight iterations (10 μg/ml) and an average PRPs-to-tetanus toxoid (TT) ratio of 1/2.6 in terms of weight (69).	In a clinical trial, 1141 infants were divided into three groups and given different vaccines. 99.7% of the infants developed antibody levels above 1 g/mL, indicating adequate protection against Hib (30).
	Hiberix		HIBERIX is made of Hib CPS and TT derived from specific strains, with CPS linked to TT through covalent bonds. TT is obtained from *Clostridium tetani*, detoxified using formaldehyde, and purified (70).	The immunogenicity of HIBERIX was evaluated in a controlled study. Anti-PRP geometric mean concentrations (GMCs) were 5.19 mcg/mL one month after the third dose of HIBERIX given at 2, 4, and 6 months of age. 81.2% of participants had anti-PRP titers above one mcg/mL (70).
	ActHIb		The vaccine consists of the Hib CPS (PRP), a high-molecular-weight polymer derived from the Hib strain 1482 cultivated in a semi-synthetic medium. This PS is covalently bound to TT (71).	Native American populations have high Hib disease rates but low response to Hib vaccines. 75% of Alaskan Native Americans had adequate antibody response after three ActHIB doses. Unvaccinated 12–24-month-olds responded well to a single ActHIB dose. ActHIB consistently shielded infants and older children from Hib disease in trials (71).
	PedvaxHib		PedvaxHIB is a preparation consisting of the refined CPS (PRP) derived from Hib, which is chemically linked to an outer membrane protein complex (OMPC) obtained from *N. meningitidis* serogroup B (72).	Vaccination with PedvaxHIB at 2 months increases antibody levels. A booster at 12 months further raises antibodies, potentially protecting against Hib. Studies compare antibody responses to different Hib vaccines. PedvaxHIB shows higher levels after one injection but not after two. PedvaxHIB’s immunogenicity is superior to ProHIBIT after one dose. Differences exist in antibody responses to PedvaxHIB and HibTITER across age groups (72).
	Sii HibPRO		Each 0.5 ml of Sii HibPRO vaccine contains at least 10 μg of PRP and 19-33 μg of TT as a carrier protein. The vaccine’s diluent is a 0.4% NaCl solution. PRP from Hib CPS is activated with CNBr, derivatised with adipic hydrazide, and then coupled to TT through carbodiimide condensation. The conjugate undergoes purification and lyophilisation. PRP has a molecular weight distribution of over 50% and less than 0.3KD, as confirmed by SEC (73).	The Sii HibPRO vaccine’s immune response and memory were satisfactory and aligned with previous literature. Safety and tolerability in infants were excellent in phase III and post-marketing studies. Adverse event frequency was comparable to WHO standards. Sii HibPRO is a safe, cost-effective, highly immunogenic vaccine. Including Hib vaccines in immunisation schemes may reduce costs and improve combat against Hib diseases (73).
	VaxemHib		Vaxem Hib is a conjugate vaccine made by Chiron Vaccines in Italy through a controlled hydrolysis process of PRP PS. Using an adipic acid spacer, the process creates OSs that are activated and linked to a carrier protein. The carrier protein in this vaccine is CRM197, a non-toxic mutant of diphtheria toxin. Aluminium hydroxide is an adjuvant in the final vaccine formulation (74).	The reactogenicity characteristics of the Hib vaccine were found to be safe with no serious adverse events. Local and systemic reactions were mild and short-lived, supporting the vaccine’s safety. Three doses of the Hib vaccine showed high immunogenicity. However, protection after two doses was insufficient, with only 58% of children adequately protected. A previous study by Kanra et al. in 2000 also stressed the need for three doses of the Vaxem Hib vaccine to ensure adequate protection for over 90% of recipients (74).
	HibTiter		Mutant diphtheria conjugate PRP-CRM or HbOC, known as HibTITER, is a vaccine using CRM197 protein carrier and low-molecular-weight PS PRP. HibTITER, created by Wyeth Pharmaceuticals Inc., was approved by the US FDA in 1990 and used until 2007 (75, 76).	The immunogenicity of the HbOC vaccine was tested on 432 infants aged 1-6 months. Infants received three doses of 10-µg HbOC at 2-month intervals. The response was seen in over 90% after two doses and over 98% after three doses, with anti-Hib CPS antibody levels ≥1 µg/ml. Long-term antibody reaction was observed, with over 80% maintaining anti-HbP levels ≥1 µg/mL at 2 years of age (77).
*N. meningitidis*	Menveo	*Meningococcal* CPS undergoes acid hydrolysis to create smaller OS fragments, isolated via chromatography for vaccine suitability. Carrier proteins like DT, CRM197, and TT from bacterial toxins are used in *N. meningitidis* vaccines. DT and CRM197 are from C. diphtheriae, while TT is from *C. tetani*. Carrier proteins activate immune responses for immune memory development. OSs and proteins in the vaccine are chemically modified for conjugation (78). Conjugate of Conjugate vaccines are complex, with various factors affecting potency. Each conjugate must undergo clinical studies for effectiveness. Different conjugates may vary in immunogenicity. Avoid vaccine interchange during multiple-dose immunisation programs (79).	MenACWY-CRM is approved in the EU for immunising individuals aged 11 and above at risk of *N. meningitidis* exposure. Each dosage includes 10 μg of serogroup A PS and 5 μg PS from each of serogroups C, W-135, and Y conjugated to around 47 μg of CRM197, a mutant of diphtheria toxin (DT) (80).	A trial compared MenACWY-CRM and MPSV4 vaccines’ immunogenicity. MenACWY-CRM had higher immunogenicity after 1 month. MenACWY-CRM recipients had higher antibody titers for serogroups A, C, and Y. MenACWY-CRM recipients maintained higher antibody titers at 12 months. MenACWY-CRM induced a robust immune response with detectable antibodies for at least 12 months (81).
	Menactra		The initial *meningococcal* quadrivalent conjugate vaccine, using DT as a carrier protein, was approved in 2005 for people aged 11-55. In 2007, it was expanded to children aged 2-10. The coverage was widened in 2011 to include children aged 9-23 months. Children aged 9-23 months receive two doses three months apart, while those aged 2-55 get one dose (82).	The MenACWY-DT vaccine was safe and effective in two doses for infants and toddlers and did not affect other vaccines. Antibody levels and efficacy decreased after 5 years, notably for serogroups C and Y. Older individuals had breakthrough cases of *meningococcal* disease. Post-licensure evaluation showed a potential risk of Guillain-Barre Syndrome. A study found no increased risk with the vaccine, but caution is advised for those with a GBS history. The CDC recommends current vaccination strategies (82).
	Nimenrix		Nimenrix is a *meningococcal* conjugate vaccine containing serogroups A, C, W135, and Y with TT as the carrier protein. It is approved in Europe as the first quadrivalent vaccine for individuals aged ‡12 months to prevent *meningococcal* disease caused by *N. meningitidis* serogroups (83).	Well-designed trials found one dose of Nimenrix induces a robust immune response against several *meningococcal* serogroups. It is well-tolerated in various age groups. Nimenrix can be safely given with routine vaccines without affecting immune responses. Limited data suggests its immunogenicity persists, but long-term studies are needed. Evidence supports a single dose of Nimenrix as beneficial in preventing *meningococcal* disease in various age groups, including 12-month-olds (83).
	Menjugate, Meningite, Meningitec		Menjugate is a conjugate vaccine using mutated CRM197 as a carrier protein for *meningococcal* serogroup C CPS (84). Meningite also contains MenC Capsular OSs and CRM197 as carrier proteins (85). Meningitec and Menjugate are vaccines with serogroup C PS conjugated to non-toxic DT derivatives from Wyeth Vaccines and Novartis Vaccines and Diagnostics (86).	A study compared immunologic memory induction between conjugated and plain *meningococcal* C PS vaccine. Toddlers given conjugate vaccine show high antibody levels and memory response. Plain vaccine is less effective and reduces immune response for a year (87).
	NeisVac-C		The serogroup C PS in NeisVac-C is first de-O-acetylated with sodium hydroxide. Then, the amino groups are reacetylated with acetic anhydride to maintain epitopes. The de-O-acetylated OS is conjugated to the TT by reductive amination. NeisVac-C contains 10 μg of *meningococcal* serogroup C OS, 10-20 μg of TT, 0.5 mg of Al (OH) and 4.1 mg of NaCl. The vaccine is presented in a single-dose, prefilled syringe without preservatives (86).	The MCC-TT vaccine showed high immunogenicity with a single dose, confirmed by bactericidal antibodies in all infants at a 100% rate. 96% of infants had a significant increase in SBA titer against the C11 strain, with a 123-fold rise. Subsequent doses of MCC led to further notable increases in C11 SBA GMT, with a 2.4-fold increase after the second dose and a 1.4-fold increase after the third dose (88).
	MenAfriVac		The Meningitis Vaccine Project (MVP) created a new vaccine to decrease African meningitis outbreaks. The vaccine, called PsA-TT, a group A *meningococcal* PS–TT conjugate vaccine, was licensed in India in 2009 and prequalified by the WHO in 2010 (89).	The use of MenAfriVac in the meningitis belt, targeting ages 1-29, significantly reduced cases. After mass campaigns, there was a decrease in meningitis cases from *N. meningitidis* serogroup A. Suspected cases were reduced by 60% in vaccinated compared to non-vaccinated. A similar reduction in districts exceeded the epidemic threshold (90).
*S. pneumoniae*	Prevnar 20	The introduction of PCV7 in 2000 reduced illness from vaccine serotypes and serotype 6A. PCV7 also lowered pneumonia, ear infections, and tube placements in children. PCV13 in 2010 decreased IPD in children and adults. PCV13 is effective in preventing IPD in those over 65. ACIP recommends PCV13 and PPSV23 for adults over 65 (91).	PCVs, unlike non-conjugated *pneumococcal* vaccines, induce a T-cell-dependent immune response, leading to strong antibodies and immunological memory. They also promote mucosal immunity, potentially reducing *S. pneumoniae* transmission. PCV20 is an extension of PCV13 with additional serotypes based on global prevalence, disease-causing potential, antibiotic resistance, and disease severity. The new serotypes covered by PCV20 account for over 30% of invasive *pneumococcal* disease cases in adults (92).	The immunogenicity of PCV20 was confirmed in clinical trials, comparing it to PCV13 and PPSV23. Immune responses against *pneumococcal* serotypes were assessed using opsonophagocytic activity assays. Adults in different age groups were randomly assigned PCV20 or PCV13, followed by PPSV23. PCV20’s immune responses were non-inferior to PCV13 and most of PPSV23, but not for one serotype. A significant increase in immune response was seen in PCV20 recipients. PCV20’s effectiveness was shown in subjects aged 18-49, 50-59, and 60-64, meeting immunobridging requirements for all 20 serotypes. In individuals aged ≥65 years, PCV20 showed strong immune responses across all 20 serotypes, regardless of prior vaccination (92).
	VAXNEUVANCE		V114 (VAXNEUVANCETM) is a *pneumococcal* conjugate vaccine consisting of 15 valences. Each 0.5 mL dose of the vaccine contains 2 mg of *pneumococcal* CPS from serotypes 1, 3, 4, 5, 6A, 7F, 9 V, 14, 18C, 19A, 19F, 23F, 22F, and 33F, along with 4 mg of serotype 6B conjugated to CRM197 carrier protein. The vaccine formulation is adjuvanted with 125 mg of aluminium phosphate (93).	V114 showed non-inferiority to PCV13 for all 13 shared serotypes with higher OPA GMTs at 30 days post-vaccination. The 95% CI lower limit for OPA GMT ratio (V114/PCV13) was above 0.5 for all shared serotypes, indicating superiority. V114 also outperformed in serotypes 22F and 33F, with OPA GMT ratio CI lower limit above 2.0 and percentage variation exceeding 10 points. Serotype 3 also met the superiority criteria, supported by IgG GMC analysis at 30 days post-vaccination and reverse cumulative distribution curves. Elevated immune responses for serotypes 3, 22F, and 33F were observed with V114, and subgroup analysis by age and demographics aligned with overall population results (93).
	Prevnar 13		PCV13 contains PSs from various serotypes conjugated to CRM197 protein, similar to PCV7, and adsorbed to aluminium phosphate. The quantity of each serotype in PCV7, except for serotype 6B, was set at 2.2 g. This quantity was used for Prevnar serotypes in PCV13 due to efficacy in studies. The dosage of 2.2 g was also chosen for the additional serotypes (94).	PCV13 induced an immune response with IgG antibodies for all 13 serotypes in the vaccine, including an extra six, with serotype 3 having the weakest IgG response. However, the OPA response to serotype 3 with PCV13 was significantly higher than with PCV7. This suggests PCV13 may offer better protection against serotype 3. In contrast, PCV7’s antibodies for serotypes 5, 6A, and 19A did not lead to strong OPA responses, indicating limited immunity from PCV7. Therefore, PCV13 triggers an immune reaction that defends against both PCV7 serotypes and the additional six in PCV13 (94).
	Prevnar		PNCRM7 (Prevnar) is a vaccination against *pneumococcus* comprising seven distinct CPS antigens derived from the bacteria *S. pneumoniae*. These antigens, namely 4, 6B, 9 V, 14, 18C, 19F, and 23F, are each linked to CRM197 (95).	The immunogenicity of PNCRM7 was demonstrated in clinical trials with healthy children of various ages. Infants received three injections at specific intervals, followed by a booster shot. Antibody levels against seven *pneumococcal* serotypes were measured after three or four doses. PNCRM7 was found to be effective in healthy infants and children in trials. A decline in antibody levels occurred before the booster dose but remained higher than before vaccination. The booster dose of PNCRM7 resulted in a significant increase in antibody levels against all seven serotypes (95).
	Synflorix		PHiD-CV contains 1 μg of each CCPS corresponding to the *pneumococcal* serotypes 1, 5, 6B, 7F, 9V, 14, and 23F, in addition to 3 μg of serotype 4, linked to a 42-kDa cell-surface lipoprotein. It also has serotype 18C CPS conjugated to TT and 19F CPS conjugated to DT. The total PS content in PHiD-CV is the same as in 7vCRM. The *pneumococcal* conjugates in the vaccine are connected to an aluminium phosphate adjuvant (96).	Infants aged 6-16 weeks, meeting specific health criteria, were chosen for the study. They received either PHiD-CV or 7vCRM in three doses at specified intervals. A booster shot was given between 11-18 months in two trials. Vaccine effectiveness was evaluated by assessing antibody levels against ten *pneumococcal* serotypes. This assessment was done a month after primary and booster vaccinations. An antibody level of 0.2 mg/mL is considered equivalent to 0.35 mg/mL without 22F-adsorption, meeting the WHO standard for comparing *pneumococcal* vaccines (97).
	Pn-MAPS24v		Pn-MAPS24v is a novel 24-valent *pneumococcal* vaccine formed using the MAPS platform, known for strong immune responses in animals. This platform uses biotin and rhizavidin to create a vaccine with 24 PS, including 13 from PCV13 and 10 from PPSV23. Serotypes like 20B are prevalent, while others like 20A are rare. Each serotype is tagged with biotin and combined with a fusion protein containing *pneumococcal* proteins. This fusion enhances immunity and helps eliminate *pneumococcal* strains (98).	In the study, the Pn-MAPS24v vaccine was well-tolerated in healthy individuals aged 18-85, showing similar safety to PCV13. Local reactions were mild and did not affect biotin levels, with effective immune responses observed for all 24 serotypes, particularly with the 5-µg dosage (98). Toddlers showed satisfactory safety profiles for Pn-MAPS24v and PCV13, with similar immune responses for common serotypes but lower responses for some due to combination vaccination schedules. Strategies are being developed to improve responses to serotype 12F, suggesting multiple doses may be needed for optimal immunity. Based on safety and immunogenicity findings for Pn-MAPS24v, further assessment in infants is encouraged (99).
	PNEUMOSIL		The *Pneumococcal* PS Conjugate Vaccine (Adsorbed) (10-valent) consists of saccharides from various *S. pneumoniae* serotypes (1, 5, 6A, 6B, 7F, 9V, 14, 19A, 19F, and 23F) attached to the CRM197 protein. The saccharides are linked using CDAP chemistry and bonded to the carrier protein CRM197 to form the glycoconjugate. The vaccine is prepared by combining the conjugates with polysorbate 20 and aluminium phosphate (100).	Indian clinical trials: The Phase 1 study in healthy adults showed safe and tolerable results, leading to African and Indian programs advancing to Phase 2; the Phase 2 study in toddlers demonstrated safety, tolerability, and immunogenicity, supporting progression to Phase 3; and the Phase 3 trial in infants achieved goals with PNEUMOSIL showing similar safety, tolerability, and immune responses to Prevnar 13 and Synflorix. African clinical trials: The Phase 1/2 trial assessed the safety and efficacy of PNEUMOSIL in various age groups, leading to the initiation of Phase 3; a Phase 3 study in The Gambia confirmed the safety, immunogenicity, and immune response persistence of PNEUMOSIL (101).
	VAX-24		VAX-24 is a liquid with CPS antigens from various *S. pneumoniae* serotypes. These include serotypes 1, 2, 3, 4, 5, 6A, 6B, 7F, 8, 9N, 9V, 10A, 11A, 12F, 14, 15B, 17F, 18C, 19A, 19F, 20B, 22F, 23F, and 33F, covering those in PCV13 and PPV23. PSs are conjugated to eCRM, a carrier protein with pAMF amino acid. This allows targeted attachment via click chemistry for conjugated compound formation. The 24 conjugated drug substances undergo sterilisation and are combined for VAX-24 potency. The primary drug substance is blended with aluminium phosphate under sterile conditions for VAX-24 DP production in single-dose vials (102).	A study in the USA compared VAX-24 and PCV20, with participants meeting specific criteria. Both vaccines showed similar safety profiles, with most participants having adverse events. VAX-24 at 2.2 μg dose had better serotype coverage than PCV20 and was non-inferior for shared serotypes (103).

## 7 Future trends in biochemistry techniques for conjugation

Biochemistry is constantly evolving, and new techniques and technologies are emerging to enhance the conjugation of bacterial vaccines further. Some future trends include:

### 7.1 GMMA and OMV

Outer Membrane Vesicles (OMVs) are naturally secreted by Gram-negative bacteria during their growth process. Through genetic modification, the yield of OMVs can be enhanced, leading to the formation of Generalised Modules for Membrane Antigens (GMMA). By introducing additional genetic alterations, the toxicity of GMMA can be adjusted by modifying the lipid A composition. GMMA exhibits a diverse array of antigens displayed in their natural form and structure, mirroring the surface of the bacteria. They possess an ideal size for triggering immune responses and possess inherent adjuvant properties as they contain Toll-like receptor (TLR) agonists. GMMA derived from Gram-negative bacteria with O-antigen chains on their surface are considered potential vaccine candidates, especially in regions with limited resources, where high production expenses may pose a barrier (31). *Shigella sonnei* (*S. sonnei*) GMMA has been subjected to clinical trials where it was found to be well tolerated and capable of eliciting an immune response (40, 41). GMMA and OMVs can be manipulated to express glycans or proteins from a pathogen of interest, making them a platform for PS vaccines. The use of OMVs and GMMA as carriers for vaccines is versatile and flexible and has been extensively studied for their safety profiles and self-adjuvant properties. Their large size and surface area make them effective in uptake by APCs, and they can be efficiently manufactured using high-yielding production platforms. Several groups have reported successful applications of OMVs and GMMA as carriers for various antigens (42). In a comparative study evaluating immunogenic response in mice, GMMA generated a higher production of anti-O-antigen IgG compared to glycoconjugate when administered without Alhydrogel. When both GMMA and glycoconjugate were formulated on Alhydrogel, they resulted in comparable levels of sustained anti-O-antigen IgG with bactericidal properties. While glycoconjugates are a widely accepted method for bacterial vaccines, their utilization can be expensive, especially in cases requiring multiple components. GMMA presents a promising alternative due to its similar immunogenicity and more straightforward production process, making it an attractive candidate for *Shigella* vaccine development (43).

### 7.2 Bioconjugation

**Figure 6:**
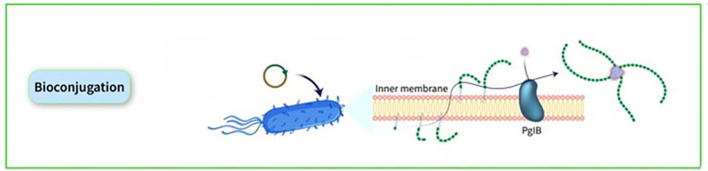
The conjugate is synthesized through a single-step process wherein the saccharide antigen and the carrier protein are expressed within *E. coli* cells and coupled *in vivo*. The formation of PS takes place on undecaprenol pyrophosphate, a lipid anchor found in the cytoplasm. Subsequently, this PS is moved to the periplasmic region, where an OTase recognizes the lipid-linked reducing end sugar and then transfers the PS to an acceptor-sequon on the carrier protein. This series of steps ultimately results in the creation of the conjugate.

Many noncovalent efforts to develop effective delivery systems combining PSs and carrier proteins have been unsuccessful. Weakly bound carrier proteins are inefficiently retrieved by BCRs, resulting in inadequate T-cell assistance for B cells. The traditional method for manufacturing licensed glycoconjugate vaccines involves chemically conjugating PSs to carrier proteins, known as ‘classical conjugates.’ PSs are typically extracted from bacterial cultures and randomly conjugated, resulting in high molecular weight, cross-linked, and heterogeneous products. In certain cases, OSs are employed, obtained through depolymerization or synthesis, allowing for more defined conjugation via reducing ends or spacers. The production of these chemically derived glycoconjugate vaccines necessitates multiple processes for both protein carriers and PSs. Recent advancements in technology for *in vivo* conjugation of PSs to proteins, termed ‘bioconjugates,’ have been made in the past two decades. This technology utilizes glycoengineered *Escherichia coli* (*E. coli*) strains to produce glycans and carrier proteins simultaneously, streamlining glycoconjugate production. The PS is synthesized on a lipid anchor, transferred to the periplasm, and coupled to carrier proteins by the oligosaccharyltransferase enzyme, resulting in the formation of bioconjugates. This method enhances production efficiency, reduces process steps, and ensures control over T-cell helper protein-specific peptides. Classical conjugates yield OSs of diverse sizes and random conjugation, potentially affecting protein epitopes. In contrast, bioconjugates benefit from a regulated saccharide-expressing system, leading to smaller, more homogeneous structures and allowing for precise sugar insertion without affecting the protein, typically resulting in 1 or 2 chains per protein molecule (32). The initial discovery of the N-linked glycosylation system in bacteria, specifically in *Campylobacter jejuni* (*C. jejuni*), occurred twenty years ago. Since then, various glycosylation systems in prokaryotic organisms have been identified, including O-linked glycosylation systems that do not have analogous counterparts in eukaryotic organisms. Subsequently, the introduction of glycosylation pathways into *E. coli* through genetic recombination gave rise to the field of bacterial glycoengineering. This emerging biotechnological tool harnesses prokaryotic glycosylation systems within *E. coli* as a host to produce recombinantly glycosylated proteins. Over the past decade, advancements in our understanding of prokaryotic glycosylation systems have led to the development of an improved glycoengineering toolbox. At present, glycoengineering employs two main approaches for the recombinant glycosylation of proteins, capable of generating N- or O-linkages: OTase-dependent and OTase-independent methods. The conjugate is synthesized through a single-step process wherein the saccharide antigen and the carrier protein are expressed within *E. coli* cells and coupled *in vivo*. The formation of PS takes place on undecaprenol pyrophosphate, a lipid anchor found in the cytoplasm. Subsequently, this PS is moved to the periplasmic region, where an OTase recognizes the lipid-linked reducing end sugar and then transfers the PS to an acceptor-sequon on the carrier protein. This series of steps ultimately results in the creation of the conjugate (**Figure 6**). In bioconjugate vaccine development, three OTases have been utilized: the widely used N-linking OTase PglB, in addition to the O-linking OTases PglL and PglS (44). a study utilizing this technique demonstrated the production and analysis of bioconjugate vaccines against the two main *hypervirulent Klebsiella pneumoniae* (*K. pneumoniae*) serotypes, K1 and K2, using genetically modified *E. coli* cells, resulting in immunogenic and effective bioconjugates that protected mice from lethal infection caused by two hvKp strains, NTUH K-2044 and ATCC 43816 (45). Over the past ten years, the immunogenicity of bioconjugate vaccines targeting various pathogens, including *Shigella dysenteriae* (*S. dysenteriae*), *S. flexneri*, *Staphylococcus aureus* (*S. aureus*), *E. coli*, *Francisella tularensis* (*F. tularensis*), *Burkholderia pseudomallei* (*B. pseudomallei*), *S. pneumoniae*, *Acinetobacter baumannii* (*A. baumannii*), and *K. pneumoniae*, has been validated in preclinical animal studies (46–54). Some bioconjugates are currently in clinical evaluation. The *S. dysenteriae* vaccine, a bioconjugate, has been tested in humans with favorable safety and immune response outcomes. It generated significant immune responses against O1 PSs and functional antibodies, demonstrating the technology’s effectiveness in maintaining both sugar and protein epitopes (55). A bioconjugate vaccine for *S. flexneri* 2a has successfully advanced through Phase-1 and Phase-2b trials, showing immunogenicity and protection post-Shigella challenge (56, 57). Additionally, ExPEC4V, a tetravalent bioconjugate vaccine against *E. coli*, has exhibited promise in two Phase-1 trials, with ongoing development by Glycovaxyn and Janssen Pharmaceuticals, Inc (58). Phase-2 trial results indicated strong immunogenicity and safety at specific antigen doses (59).

Certain microorganisms, such as *K. pneumoniae* strain ATCC 25955, have natural advantages over model microorganisms like *E. coli* in terms of growth rate, protein expression, and metabolic pathways. These advantages make *K. pneumoniae* strain ATCC 25955 a suitable strain for glycoengineering. In a study, two glycoengineered *K. pneumoniae* strains with different O-antigen serotypes were constructed and successfully used to produce glycoprotein-bearing nanovaccines. This research offers a successful remedy for developing nanovaccines against *K. pneumoniae* and has potential implications for future synthetic biological research (60).

The effectiveness of a glycoconjugate vaccine is influenced by factors including sugar length, sugar load, and binding interactions affecting immunogenicity. A recent review concluded that optimal glycan chain length and epitope density are crucial for vaccine efficacy in glycoconjugates (61). Classical conjugates can accommodate multiple saccharide chains, while bioconjugates face challenges in incorporating similar quantities. A reduced number of saccharide chains per protein may result in diminished immunogenicity due to lower BCR crosslinking capacity. Although traditional methods are more complex than bioconjugation, bioconjugate technology still faces challenges, particularly in optimizing *E. coli* as a production model. Key technical challenges include enhancing the yield of oligosaccharyltransferase to ensure effective glycosylation of protein consensus sequences. Screening oligosaccharyltransferase mutants may improve saccharide occupancy and increase antigen production yield. Due to their unique properties, bioconjugate vaccines can be likened to traditional conjugates formed by linking OSs to carrier proteins at the sugar’s reducing end, with clinical trials conducted on two bioconjugate vaccines against *S. pneumoniae* and *S. flexneri* 2a, both of which have also been examined with classical conjugates in separate studies (32).

### 7.3 Nanoparticles

Qβ virus-like particles (VLPs) have been evaluated as a vehicle for conveying brief synthetic *S. pneumoniae* serotype 3 and 14 OSs via copper-catalyzed click chemistry, demonstrating their capacity to induce serotype-specific, safeguarding, and enduring IgG antibodies of nanomolar affinity in mice (62).

Gold nanoparticles have also been utilized in numerous attempts to produce glycoconjugate vaccines. These nanoparticles possess the ability to undergo functionalization due to the robust interaction between an Au atom and sulfur, thereby allowing for the incorporation of a synthetic tetrasaccharide epitope of *S. pneumoniae* serotype 14, a T-helper ovalbumin 323–339 peptide and D-glucose. Studies conducted on animal models have demonstrated that these glyconanoparticles can elicit a specific functional antibody response to the saccharide (63, 64). Additionally, a recent investigation employed gold nanoparticles decorated with synthetic OSs of *S. pneumoniae* serotypes 19F and 14, examining the impact of simultaneously displaying two carbohydrate epitopes from different bacterial serotypes on the immune response (63–65).

Nanoparticles have been the subject of extensive investigation within the realm of vaccine research; however, their function as carriers in the development of glycoconjugate vaccines has not been thoroughly examined, which restricts the comprehension of the optimal characteristics necessary for the effective induction of immune responses. The researchers concentrated on two self-assembling proteins: the Hcp1cc protein, which assembles into ring-shaped hexamers capable of oligomerizing into nanotubes, and *Helicobacter pylori* ferritin (Hpf), which yields spherical nanoparticles. Both proteins were conjugated with MenW OSs owing to the presence of surface lysines, resulting in elevated levels of glycosylation. Characterization methodologies, including Asymmetrical Flow Field-Flow Fractionation (AF4), validated the successful conjugation of nanoparticles. The glycoconjugated nanoparticles were administered to mice utilizing an AS01-adjuvanted regimen, with MenW-CRM197 serving as the control vaccine. The results indicated that the MenW-Ferritin conjugate elicited a more robust immune response and enhanced bactericidal activity in comparison to other MenW-nanoparticles. Importantly, the dimensions of the nanotubes did not exert a significant effect on the immune response, whereas the spherical ferritin nanoparticles exhibited the highest levels of immunogenicity. These findings are consistent with previous studies concerning inorganic nanoparticles, which demonstrate that spherical nanoparticles facilitate cellular uptake, activate APCs, and promote trafficking to lymph nodes, rendering them more efficacious for immune activation than rod-shaped counterparts. This effectiveness is posited to arise from a reduced energy expenditure required for the cellular internalization of spherical morphologies and an optimal contact area with cell membranes, thereby enhancing dendritic cell (DC) uptake and Th1 immune responses. This investigation emphasizes the promise of protein-based nanoparticles, particularly Hpf, in the advancement of glycoconjugate vaccines. In contrast to inorganic nanoparticles, protein scaffolds such as ferritin may display a variety of T-cell epitopes, potentially amplifying the immune response. These findings underscore the notion that while the shape of nanoparticles exerts a more pronounced influence than size, protein nanoparticles represent a promising strategy for developing novel glycoconjugate vaccines targeting bacterial pathogens (66).

## 8 Discussion

A dense layer of OSs and PSs envelop microbial pathogens’ surfaces, enhancing their ability to adhere to host cells and initiate invasion. Consequently, these exposed sugar molecules are attractive targets for use as antigens in the development of vaccines. Moreover, immunogenicity can be enhanced by conjugating PSs to a carrier protein, producing glycoconjugate antigens that stimulate B cell maturation and shift antibody classes. Thus, glycoconjugation plays a crucial role in the formulation of carbohydrate-based vaccines. The effectiveness of the conjugation process, the saccharide-to-protein ratio, and the characteristics of the resulting conjugate, all of which impact the immunogenicity of the vaccine, are significantly influenced by the chemistry of the conjugation reactions. Additionally, thorough biological investigations are necessary to assess the immunogenic potential of completely homogeneous conjugates, as the immune response to carbohydrate-protein conjugates is modulated by various factors, including the size of the carbohydrate epitope, the carrier protein, the nature and quantity of covalent linkages, and the composition of the linker molecule. Recent advancements have led to significant progress in techniques aimed at simplifying and expediting the production of glycoconjugates. The chemical and enzymatic approaches discussed in this paper present numerous opportunities for researchers to develop customised glycoconjugate vaccines. However, in certain instances, a combination of methods may be essential to facilitate the synthesis of glycoconjugates effectively. The progression in bacterial glycoengineering, nanoparticle formulation, and bioconjugation techniques is poised to transform glycoconjugate vaccines. Integrating these innovations may yield effective, scalable, and economical vaccines for priority pathogens in resource-limited areas. Investigating the synergistic effects of these technologies could significantly advance the global fight against bacterial diseases, enhancing vaccine accessibility and public health outcomes. In summary, advancements in glycoconjugate vaccine development are notable, yet limitations persist. Glycoconjugate purification complexity hinders high yield and cost-effective production, particularly in resource-limited regions. Furthermore, the immunogenic mechanisms in various populations, especially younger individuals, remain inadequately understood, obstructing effective vaccine design. Although production techniques have improved, their labour-intensive nature increases costs and limits scalability. Conventional conjugation methods frequently produce heterogeneous, high-molecular-weight products, potentially affecting vaccine consistency and efficacy. Overcoming these challenges necessitates ongoing innovation in purification, conjugation, and mechanistic research to develop accessible, effective, and scalable glycoconjugate vaccines.

## References

[1] Mukhopadhyay B Maurer SV Rudolph N van Well RM Russell DA Field RA. From solution phase to “On-column” Chemistry: Trichloroacetimidate-based glycosylation promoted by perchloric acid–silica. J Organic Chem. (2005) 70:9059–62. doi: 10.1021/jo051390g 16238354

[2] Bahonar S Ghazvinian M Haghshenas MR Goli HR Mirzaei B. Purification of PIA and rSesC as Putative Vaccine Candidates Against Staphylococcus aureus. Rep Biochem Mol Biol. (2019) 8:161–7. PMC6844615 31832440

[3] Gholami SA Goli HR Haghshenas MR Mirzaei B. Evaluation of polysaccharide intercellular adhesion (PIA) and glycerol teichoic acid (Gly-TA) arisen antibodies to prevention of biofilm formation in Staphylococcus aureus and Staphylococcus epidermidis strains. BMC Res Notes. (2019) 12:691. doi: 10.1186/s13104-019-4736-8 31653277 PMC6815028

[4] Mirzaei B Babaei R Haghshenas MR Mohammadi F Homayoni P Shafaei E. PIA and rSesC Mixture Arisen Antibodies Could Inhibit the Biofilm-Formation in Staphylococcus aureus. Rep Biochem Mol Biol. (2021) 10:1–12. doi: 10.52547/rbmb.10.1.1 34277863 PMC8279720

[5] Zhu H Rollier CS Pollard AJ. Recent advances in lipopolysaccharide-based glycoconjugate vaccines. Expert Rev Vaccines. (2021) 20:1515–38. doi: 10.1080/14760584.2021.1984889 34550840

[6] Mirzaei B Babaei R Valinejad S. Staphylococcal Vaccine Antigens related to biofilm formation. Hum Vaccin Immunother. (2021) 17:293–303. doi: 10.1080/21645515.2020.1767449 32498595 PMC7872035

[7] Mirzaei B Babaei R Zeighami H Dadar M Soltani A. Staphylococcus aureus putative vaccines based on the virulence factors: A mini-review. Front Microbiol. (2021) 12:704247. doi: 10.3389/fmicb.2021.704247 34539603 PMC8447878

[8] Mirzaei B Moosavi SF Babaei R Siadat SD Vaziri F Shahrooei M. Purification and evaluation of polysaccharide intercellular adhesion (PIA) antigen from staphylococcus epidermidis. Curr Microbiol. (2016) 73:611–7. doi: 10.1007/s00284-016-1098-5 27460584

[9] Mirzaei B Mousavi SF Babaei R Bahonar S Siadat SD Shafiee Ardestani M. Synthesis of conjugated PIA-rSesC and immunological evaluation against biofilm-forming Staphylococcus epidermidis. J Med Microbiol. (2019) 68:791–802. doi: 10.1099/jmm.0.000910 30990402

[10] Pace D. Glycoconjugate vaccines. Expert Opin Biol Ther. (2013) 13:11–33. doi: 10.1517/14712598.2012.725718 22992106

[11] Micoli F Del Bino L Alfini R Carboni F Romano MR Adamo R. Glycoconjugate vaccines: current approaches towards faster vaccine design. Expert Rev Vaccines. (2019) 18:881–95. doi: 10.1080/14760584.2019.1657012 31475596

[12] Takagi M Lima RB Albani SMF Zangirolami TC Tanizaki MM Cabrera-Crespo J. Purification of capsular polysaccharide produced by Haemophilus influenzae type b through a simple, efficient and suitable method for scale-up. J Ind Microbiol Biotechnol. (2008) 35:1217–22. doi: 10.1007/s10295-008-0428-4 18712545

[13] Gonçalves V Takagi M Carmo T Albani S Pinto J Zangirolami T. Communicating current research and educational topics and trends in applied microbiology. (2007) 1:450–7.

[14] Albani SMF da Silva MR Takagi M Cabrera-Crespo J. Improvement in the Purification Process of the Capsular Polysaccharide from Haemophilus influenzae Type b by Using Tangential Ultrafiltration and Diafiltration. Appl Biochem Biotechnol. (2012) 167:2068–75. doi: 10.1007/s12010-012-9750-4 22665219

[15] Braga LG Cabrera-Crespo J Takagi M. Cell separation of Haemophilus influenzae type b through tangential microfiltration. Separation Purification Technology. (2021) 257:117965. doi: 10.1016/j.seppur.2020.117965

[16] Suarez N Massaldi H Fraguas LF Ferreira F. Improved conjugation and purification strategies for the preparation of protein–polysaccharide conjugates. J Chromatogr A. (2008) 1213:169–75. doi: 10.1016/j.chroma.2008.10.030 18992885

[17] Morais V Dee V Suárez N. Purification of capsular polysaccharides of streptococcus pneumoniae: traditional and new methods. Front Bioengineering Biotechnol. (2018) 6. doi: 10.3389/fbioe.2018.00145 PMC6194195 30370268

[18] Macha C Lavanya A Nanna R. Purification of Streptococcus pneumoniae capsular polysaccharides using aluminium phosphate and ethanol. Int J Pharm Pharm Sci. (2014) 6:664–70.

[19] Yuan Y Ruppen M Sun W-Q Chu L Simpson J Patch J. Shortened Purification Process for the Production of Capsular Streptococcus Pneumoniae Polysaccharides. United States: Google Patents (2014).

[20] Zanardo RT Ferri A Figueiredo DB Kraschowetz S Cabrera-Crespo J Gonçalves V. Development of a new process for purification of capsular polysaccharide from Streptococcus pneumoniae serotype 14. Braz J Chem Engineering. (2016) 33:435–43. doi: 10.1590/0104-6632.20160333s20150140

[21] Emami P Motevalian SP Pepin E Zydney AL. Purification of a conjugated polysaccharide vaccine using tangential flow diafiltration. Biotechnol Bioengineering. (2019) 116:591–7. doi: 10.1002/bit.v116.3 30450582

[22] Lee C Chun HJ Park M Kim RK Whang YH Choi SK. Quality improvement of capsular polysaccharide in streptococcus pneumoniae by purification process optimization. Front Bioengineering Biotechnol. (2020) 8. doi: 10.3389/fbioe.2020.00039 PMC7011675 32117921

[23] Li Y Cao X Huang X Liu Y Wang J Jin Q. Novel manufacturing process of pneumococcal capsular polysaccharides using advanced sterilization methods. Front Bioengineering Biotechnol. (2024) 12. doi: 10.3389/fbioe.2024.1451881 PMC11335687 39170064

[24] Sharma S Hanif S Kumar N Joshi N Rana R Dalal J. Rapid processes for purification of capsular polysaccharides from Neisseria meningitidis serogroups A and C. Biologicals. (2015) 43:383–9. doi: 10.1016/j.biologicals.2015.06.003 26123432

[25] Avery OT Goebel WF. Chemo-immunological studies on conjugated carbohydrate-proteins : II. immunological specificity of synthetic sugar-protein antigens. J Exp Med. (1929) 50:533–50. doi: 10.1084/jem.50.4.533 PMC2131643 19869645

[26] Macleod CM Hodges RG Heidelberger M Bernhard WG. Prevention of pneumococcal pneumonia by immunization with specific capsular polysaccharides. J Exp Med. (1945) 82:445–65. doi: 10.1084/jem.82.6.445 PMC2135567 19871511

[27] Schneerson R Barrera O Sutton A Robbins JB. Preparation, characterization, and immunogenicity of Haemophilus influenzae type b polysaccharide-protein conjugates. J Exp Med. (1980) 152:361–76. doi: 10.1084/jem.152.2.361 PMC2185954 6967514

[28] Parke JC Jr. Capsular polysaccharide of Haemophilus influenzae type b as a vaccine. Pediatr Infect Dis J. (1987) 6:795–8. doi: 10.1097/00006454-198708000-00040 3313244

[29] Peltola H Kilpi T Anttila M. Rapid disappearance of Haemophilus influenzae type b meningitis after routine childhood immunisation with conjugate vaccines. Lancet (London England). (1992) 340:592–4. doi: 10.1016/0140-6736(92)92117-X 1355165

[30] Verez-Bencomo V Fernández-Santana V Hardy E Toledo ME Rodríguez MC Heynngnezz L. A Synthetic Conjugate Polysaccharide Vaccine Against Haemophilus influenzae Type b. Science. (2004) 305:522–5. doi: 10.1126/science.1095209 15273395

[31] Berti F Micoli F. Improving efficacy of glycoconjugate vaccines: from chemical conjugates to next generation constructs. Curr Opin Immunol. (2020) 65:42–9. doi: 10.1016/j.coi.2020.03.015 32361591

[32] Romano MR Berti F Rappuoli R. Classical-and bioconjugate vaccines: comparison of the structural properties and immunological response. Curr Opin Immunol. (2022) 78:102235. doi: 10.1016/j.coi.2022.102235 35988326

[33] Nguyen B Tolia NH. Protein-based antigen presentation platforms for nanoparticle vaccines. NPJ Vaccines. (2021) 6:70. doi: 10.1038/s41541-021-00330-7 33986287 PMC8119681

[34] Brisse M Vrba SM Kirk N Liang Y Ly H. Emerging concepts and technologies in vaccine development. Front Immunol. (2020) 11. doi: 10.3389/fimmu.2020.583077 PMC7554600 33101309

[35] Laera D Scarpellini C Tavarini S Baudner B Marcelli A Pergola C. Maturation of aluminium adsorbed antigens contributes to the creation of homogeneous vaccine formulations. Vaccines (Basel). (2023) 11. doi: 10.3390/vaccines11010155 PMC9862877 36680000

[36] Biemans R Micoli F Romano MR. 8 - Glycoconjugate vaccines, production and characterization. In: Rauter AP Christensen BE Somsák L Kosma P Adamo R, editors. Recent Trends in Carbohydrate Chemistry. The Netherlands: Elsevier (2020). p. 285–313.

[37] van der Put RMF Smitsman C de Haan A Hamzink M Timmermans H Uittenbogaard J. The first-in-human synthetic glycan-based conjugate vaccine candidate against shigella. ACS Cent Science. (2022) 8:449–60. doi: 10.1021/acscentsci.1c01479 PMC9088300 35559427

[38] Adamo R. Advancing homogeneous antimicrobial glycoconjugate vaccines. Accounts Chem Res. (2017) 50:1270–9. doi: 10.1021/acs.accounts.7b00106 28463499

[39] Oldrini D Fiebig T Romano MR Proietti D Berger M Tontini M. Combined chemical synthesis and tailored enzymatic elongation provide fully synthetic and conjugation-ready neisseria meningitidis serogroup X vaccine antigens. ACS Chem Biol. (2018) 13:984–94. doi: 10.1021/acschembio.7b01057 29481045

[40] Launay O Lewis DJM Anemona A Loulergue P Leahy J Sciré AS. Safety profile and immunologic responses of a novel vaccine against shigella sonnei administered intramuscularly, intradermally and intranasally: results from two parallel randomized phase 1 clinical studies in healthy adult volunteers in Europe. EBioMedicine. (2017) 22:164–72. doi: 10.1016/j.ebiom.2017.07.013 PMC5552227 28735965

[41] Obiero CW Ndiaye AGW Sciré AS Kaunyangi BM Marchetti E Gone AM. A Phase 2a Randomized Study to Evaluate the Safety and Immunogenicity of the 1790GAHB Generalized Modules for Membrane Antigen Vaccine against Shigella sonnei Administered Intramuscularly to Adults from a Shigellosis-Endemic Country. Front Immunol. (2017) 8:1884. doi: 10.3389/fimmu.2017.01884 29375556 PMC5763125

[42] van der Put RMF Metz B Pieters RJ. Carriers and antigens: new developments in glycoconjugate vaccines. Vaccines. (2023) 11:219. doi: 10.3390/vaccines11020219 36851097 PMC9962112

[43] Raso MM, Gasperini G, Alfini R, Schiavo F, Aruta MG, Carducci M, et al. GMMA and Glycoconjugate Approaches Compared in Mice for the Development of a Vaccine against Shigella flexneri Serotype 6. Vaccines (Basel). (2020) 8. doi: 10.3390/vaccines8020160 PMC7349896 32260067

[44] Harding CM Feldman MF. Glycoengineering bioconjugate vaccines, therapeutics, and diagnostics in E. coli. Glycobiology. (2019) 29:519–29. doi: 10.1093/glycob/cwz031 PMC6583762 30989179

[45] Feldman MF Mayer Bridwell AE Scott NE Vinogradov E McKee SR Chavez SM. A promising bioconjugate vaccine against hypervirulent Klebsiella pneumoniae. Proc Natl Acad Sci. (2019) 116:18655–63. doi: 10.1073/pnas.1907833116 PMC6744904 31455739

[46] Wetter M Kowarik M Steffen M Carranza P Corradin G Wacker M. Engineering, conjugation, and immunogenicity assessment of Escherichia coli O121 O antigen for its potential use as a typhoid vaccine component. Glycoconjugate J. (2013) 30:511–22. doi: 10.1007/s10719-012-9451-9 23053636

[47] Cuccui J Thomas RM Moule MG D'Elia RV Laws TR Mills DC. Exploitation of bacterial N-linked glycosylation to develop a novel recombinant glycoconjugate vaccine against Francisella tularensis. Open Biol. (2013) 3:130002. doi: 10.1098/rsob.130002 23697804 PMC3866875

[48] Garcia-Quintanilla F Iwashkiw JA Price NL Stratilo C Feldman MF. Production of a recombinant vaccine candidate against Burkholderia pseudomallei exploiting the bacterial N-glycosylation machinery. Front Microbiol. (2014) 5. doi: 10.3389/fmicb.2014.00381 PMC4114197 25120536

[49] Wacker M Wang L Kowarik M Dowd M Lipowsky G Faridmoayer A. Prevention of Staphylococcus aureus Infections by Glycoprotein Vaccines Synthesized in Escherichia coli. J Infect Diseases. (2013) 209:1551–61. doi: 10.1093/infdis/jit800 PMC3997581 24308931

[50] van den Dobbelsteen GPJM Faé KC Serroyen J van den Nieuwenhof IM Braun M Haeuptle MA. Immunogenicity and safety of a tetravalent E. coli O-antigen bioconjugate vaccine in animal models. Vaccine. (2016) 34:4152–60. doi: 10.1016/j.vaccine.2016.06.067 27395567

[51] Reglinski M Ercoli G Plumptre C Kay E Petersen FC Paton JC. A recombinant conjugated pneumococcal vaccine that protects against murine infections with a similar efficacy to Prevnar-13. NPJ Vaccines. (2018) 3:53. doi: 10.1038/s41541-018-0090-4 30393571 PMC6208403

[52] Harding CM Nasr MA Scott NE Goyette-Desjardins G Nothaft H Mayer AE. A platform for glycoengineering a polyvalent pneumococcal bioconjugate vaccine using E. coli as host. Nat Commun. (2019) 10:891. doi: 10.1038/s41467-019-08869-9 30792408 PMC6385209

[53] Aceil J Paschall AV Knoot CJ Robinson LS Scott NE Feldman MF. Immunogenicity and protective efficacy of a prototype pneumococcal bioconjugate vaccine. Vaccine. (2022) 40:6107–13. doi: 10.1016/j.vaccine.2022.09.018 PMC10388713 36115800

[54] Marshall LE Nelson M Davies CH Whelan AO Jenner DC Moule MG. An O-antigen glycoconjugate vaccine produced using protein glycan coupling technology is protective in an inhalational rat model of tularemia. J Immunol Res. (2018) 2018:8087916. doi: 10.1155/2018/8087916 30622981 PMC6304830

[55] Hatz CFR Bally B Rohrer S Steffen R Kramme S Siegrist C-A. Safety and immunogenicity of a candidate bioconjugate vaccine against Shigella dysenteriae type 1 administered to healthy adults: A single blind, partially randomized Phase I study. Vaccine. (2015) 33:4594–601. doi: 10.1016/j.vaccine.2015.06.102 26162850

[56] Riddle MS Kaminski RW Di Paolo C Porter CK Gutierrez RL Clarkson KA. Safety and Immunogenicity of a Candidate Bioconjugate Vaccine against Shigella flexneri 2a Administered to Healthy Adults: a Single-Blind, Randomized Phase I Study. Clin Vaccine Immunol CVI. (2016) 23:908–17. doi: 10.1128/CVI.00224-16 PMC5139601 27581434

[57] Talaat KR Alaimo C Martin P Bourgeois AL Dreyer AM Kaminski RW. Human challenge study with a Shigella bioconjugate vaccine: Analyses of clinical efficacy and correlate of protection. EBioMedicine. (2021) 66:103310. doi: 10.1016/j.ebiom.2021.103310 33862589 PMC8054157

[58] Huttner A Hatz C van den Dobbelsteen G Abbanat D Hornacek A Frölich R. Safety, immunogenicity, and preliminary clinical efficacy of a vaccine against extraintestinal pathogenic Escherichia coli in women with a history of recurrent urinary tract infection: a randomised, single-blind, placebo-controlled phase 1b trial. Lancet Infect Diseases. (2017) 17:528–37. doi: 10.1016/S1473-3099(17)30108-1 28238601

[59] Frenck RW Ervin J Chu L Abbanat D Spiessens B Go O. Safety and immunogenicity of a vaccine for extra-intestinal pathogenic Escherichia coli (ESTELLA): a phase 2 randomised controlled trial. Lancet Infect Diseases. (2019) 19:631–40. doi: 10.1016/S1473-3099(18)30803-X 31079947

[60] Liu Y Li S Guo Y Li X Zhu L Wang H. Genetic engineering of klebsiella pneumoniae ATCC 25955 for bioconjugate vaccine applications. Microorganisms. (2023) 11:1321. doi: 10.3390/microorganisms11051321 37317295 PMC10222708

[61] Anish C Beurret M Poolman J. Combined effects of glycan chain length and linkage type on the immunogenicity of glycoconjugate vaccines. NPJ Vaccines. (2021) 6:150. doi: 10.1038/s41541-021-00409-1 34893630 PMC8664855

[62] Polonskaya Z Deng S Sarkar A Kain L Comellas-Aragones M McKay CS. T cells control the generation of nanomolar-affinity anti-glycan antibodies. J Clin Invest. (2017) 127:1491–504. doi: 10.1172/JCI91192 PMC5373877 28287405

[63] Safari D Marradi M Chiodo F Th Dekker HA Shan Y Adamo R. Gold nanoparticles as carriers for a synthetic Streptococcus pneumoniae type 14 conjugate vaccine. Nanomedicine (London England). (2012) 7:651–62. doi: 10.2217/nnm.11.151 22630149

[64] Vetro M Safari D Fallarini S Salsabila K Lahmann M Penadés S. Preparation and immunogenicity of gold glyco-nanoparticles as antipneumococcal vaccine model. Nanomedicine (London England). (2017) 12:13–23. doi: 10.2217/nnm-2016-0306 27879152

[65] Shen Y Hao T Ou S Hu C Chen L. Applications and perspectives of nanomaterials in novel vaccine development. MedChemComm. (2018) 9:226–38. doi: 10.1039/C7MD00158D PMC6083789 30108916

[66] Dolce M Proietti D Principato S Giusti F Adamo GM Favaron S. Impact of protein nanoparticle shape on the immunogenicity of antimicrobial glycoconjugate vaccines. Int J Mol Sci. (2024) 25:3736. doi: 10.3390/ijms25073736 38612547 PMC11011275

[67] Zarei AE Almehdar HA Redwan EM. Hib vaccines: past, present, and future perspectives. J Immunol Res. (2016) 2016:7203587. doi: 10.1155/2016/7203587 26904695 PMC4745871

[68] Jones C. Vaccines based on the cell surface carbohydrates of pathogenic bacteria. Anais da Academia Bras Ciencias. (2005) 77:293–324. doi: 10.1590/S0001-37652005000200009 15895165

[69] Toraño G Toledo ME Baly A Fernandez-Santana V Rodriguez F Alvarez Y. Phase I Clinical Evaluation of a Synthetic Oligosaccharide-Protein Conjugate Vaccine against Haemophilus influenzae Type b in Human Adult Volunteers. Clin Vaccine Immunol. (2006) 13:1052–6. doi: 10.1128/CVI.00144-06 PMC1563574 16960118

[70] Briere EC. Food and Drug Administration approval for use of Hiberix as a 3-dose primary Haemophilus influenzae type b (Hib) vaccination series. MMWR Morbidity Mortality Weekly Rep. (2016) 65:418–9. doi: 10.15585/mmwr.mm6516a3 27124887

[71] AvP D. Haemophilus b conjugate vaccine (Tetanus toxoid conjugate) actHIB®. 7(8):9–10. facilities.

[72] Ahonkhai V Lukacs L Jonas L Calandra G. Clinical experience with PedvaxHIB, a conjugate vaccine of Haemophilus influenzae type b polysaccharide—Neisseria meningitidis outer membrane protein. Vaccine. (1991) 9:S38–41. doi: 10.1016/0264-410X(91)90180-E 1891956

[73] Sharma HJ Multani AS Dutta AK Joshi SM Malik S Bhardwaj S. Safety and immunogenicity of an indigenously developed Haemophilus influenzae type b conjugate vaccine through various phases of clinical trials. Hum Vaccines. (2009) 5:483–7. doi: 10.4161/hv.8582 19395868

[74] Matjila MJ Phohu TC Banzhoff A Viviani S Hoosen AA Bianchini M. Safety and immunogenicity of two Haemophilus influenzae type b conjugate vaccines. South Afr Med J. (2004) 94:43–6. 14971232

[75] Khatuntseva EA Nifantiev NE. Glycoconjugate Vaccines for Prevention of Haemophilus influenzae Type b Diseases. Russian J bioorganic Chem. (2021) 47:26–52. doi: 10.1134/S1068162021010106 PMC7980804 33776394

[76] Peltola H Aavitsland P Hansen KG Jónsdóttir KE Nøkleby H Romanus V. Perspective: A Five-Country Analysis of the Impact of Four Different Haemophilus influenzae Type b Conjugates and Vaccination Strategies in Scandinavia. J Infect Diseases. (1999) 179:223–9. doi: 10.1086/jid.1999.179.issue-1 9841843

[77] Madore DV Johnson CL Phipps DC Popejoy LA Eby R Smith DH. Safety and immunologic response to haemophilus influenzae type b oligosaccharide-CRM197 conjugate vaccine in 1- to 6-month-old infants. Pediatrics. (1990) 85:331–7. 2304786

[78] McCarthy PC Sharyan A Sheikhi Moghaddam L. Meningococcal vaccines: current status and emerging strategies. Vaccines. (2018) 6:12. doi: 10.3390/vaccines6010012 29495347 PMC5874653

[79] Bröker M Berti F Costantino P. Factors contributing to the immunogenicity of meningococcal conjugate vaccines. Hum Vaccines Immunotherapeutics. (2016) 12:1808–24. doi: 10.1080/21645515.2016.1153206 PMC4964817 26934310

[80] Cooper B DeTora L Stoddard J. Menveo®: a novel quadrivalent meningococcal CRM197 conjugate vaccine against serogroups A, C, W-135 and Y. Expert Rev Vaccines. (2011) 10:21–33. doi: 10.1586/erv.10.147 21162617

[81] Jackson LA Jacobson RM Reisinger KS Anemona A Danzig LE Dull PM. A randomized trial to determine the tolerability and immunogenicity of a quadrivalent meningococcal glycoconjugate vaccine in healthy adolescents. Pediatr Infect Dis J. (2009) 28:86–91. doi: 10.1097/INF.0b013e31818a0237 19116603

[82] Hedari CP Khinkarly RW Dbaibo GS. Meningococcal serogroups A, C, W-135, and Y tetanus toxoid conjugate vaccine: a new conjugate vaccine against invasive meningococcal disease. Infection Drug resistance. (2014) 7:85–99. doi: 10.2147/IDR.S36243 24729718 PMC3979687

[83] Croxtall JD Dhillon S. Meningococcal quadrivalent (serogroups A, C, W135 and Y) tetanus toxoid conjugate vaccine (Nimenrix™). Drugs. (2012) 72:2407–30. doi: 10.2165/11209580-000000000-00000 23231026

[84] Aaberge IS Oster P Helland OS Kristoffersen A-C Ypma E Høiby EA. Combined administration of meningococcal serogroup B outer membrane vesicle vaccine and conjugated serogroup C vaccine indicated for prevention of meningococcal disease is safe and immunogenic. Clin Vaccine Immunol. (2005) 12:599–605. doi: 10.1128/CDLI.12.5.599-605.2005 PMC1112071 15879021

[85] Rohokale R Guo Z. Development in the concept of bacterial polysaccharide repeating unit-based antibacterial conjugate vaccines. ACS Infect diseases. (2023) 9:178–212. doi: 10.1021/acsinfecdis.2c00559 36706246 PMC9930202

[86] Borrow R Findlow J. Prevention of meningococcal serogroup C disease by NeisVac-C™. Expert Rev Vaccines. (2009) 8:265–79. doi: 10.1586/14760584.8.3.265 19249967

[87] MacDonald NE Halperin SA Law BJ Forrest B Danzig LE Granoff DM. Induction of immunologic memory by conjugated vs plain meningococcal C polysaccharide vaccine in toddlers: a randomized controlled trial. Jama. (1998) 280:1685–9. doi: 10.1001/jama.280.19.1685 9832000

[88] Richmond P Borrow R Findlow J Martin S Thornton C Cartwright K. Evaluation of de-O-acetylated meningococcal C polysaccharide-tetanus toxoid conjugate vaccine in infancy: reactogenicity, immunogenicity, immunologic priming, and bactericidal activity against O-acetylated and de-O-acetylated serogroup C strains. Infection Immunity. (2001) 69:2378–82. doi: 10.1128/IAI.69.4.2378-2382.2001 PMC98168 11254596

[89] Diomandé FVK Djingarey MH Daugla DM Novak RT Kristiansen PA Collard J-M. Public health impact after the introduction of PsA-TT: the first 4 years. Clin Infect Dis. (2015) 61:S467–S72. doi: 10.1093/cid/civ499 PMC4639484 26553676

[90] Trotter CL Lingani C Fernandez K Cooper LV Bita A Tevi-Benissan C. Impact of MenAfriVac in nine countries of the African meningitis belt, 2010–15: an analysis of surveillance data. Lancet Infect diseases. (2017) 17:867–72. doi: 10.1016/S1473-3099(17)30301-8 28545721

[91] Yildirim I Shea KM Pelton SI. Pneumococcal disease in the era of pneumococcal conjugate vaccine. Infect Dis Clinics. (2015) 29:679–97. doi: 10.1016/j.idc.2015.07.009 PMC4662776 26610421

[92] Shirley M. 20-valent pneumococcal conjugate vaccine: A review of its use in adults. Drugs. (2022) 82:989–99. doi: 10.1007/s40265-022-01733-z 35793027

[93] Platt HL Cardona JF Haranaka M Schwartz HI Perez SN Dowell A. A phase 3 trial of safety, tolerability, and immunogenicity of V114, 15-valent pneumococcal conjugate vaccine, compared with 13-valent pneumococcal conjugate vaccine in adults 50 years of age and older (PNEU-AGE). Vaccine. (2022) 40:162–72. doi: 10.1016/j.vaccine.2021.08.049 34507861

[94] Gruber WC Scott DA Emini EA. Development and clinical evaluation of Prevnar 13, a 13-valent pneumocococcal CRM197 conjugate vaccine. Ann New York Acad Sci. (2012) 1263:15–26. doi: 10.1111/j.1749-6632.2012.06673.x 22830997

[95] Darkes MJ Plosker GL. Pneumococcal Conjugate Vaccine (Prevnar™ 1; PNCRM7) A Review of its Use in the Prevention of Streptococcus pneumoniae Infection. Pediatr Drugs. (2002) 4:609–30. doi: 10.2165/00128072-200204090-00005 12175274

[96] Prymula R Schuerman L. 10-valent pneumococcal nontypeable Haemophilus influenzae PD conjugate vaccine: Synflorix™. Expert Rev Vaccines. (2009) 8:1479–500. doi: 10.1586/erv.09.113 19863240

[97] Croxtall JD Keating GM. Pneumococcal polysaccharide protein D-conjugate vaccine (Synflorix™; PhiD-CV). Pediatr Drugs. (2009) 11:349–57. doi: 10.2165/11202760-000000000-00000 19725600

[98] Chichili GR Smulders R Santos V Cywin B Kovanda L Van Sant C. Phase 1/2 study of a novel 24-valent pneumococcal vaccine in healthy adults aged 18 to 64 years and in older adults aged 65 to 85 years. Vaccine. (2022) 40:4190–8. doi: 10.1016/j.vaccine.2022.05.079 35690500

[99] Borys D Rupp R Smulders R Chichili GR Kovanda LL Santos V. Safety, tolerability and immunogenicity of a novel 24-valent pneumococcal vaccine in toddlers: A phase 1 randomized controlled trial. Vaccine. (2024) 42(10):2560–71. doi: 10.1016/j.vaccine.2024.02.001 38360475

[100] Organization WH. Considerations for pneumococcal conjugate vaccine (PCV) product choice. (2021).

[101] Alderson MR Sethna V Newhouse LC Lamola S Dhere R. Development strategy and lessons learned for a 10-valent pneumococcal conjugate vaccine (PNEUMOSIL®). Hum Vaccines Immunotherapeutics. (2021) 17:2670–7. doi: 10.1080/21645515.2021.1874219 PMC8475595 33625961

[102] Fairman J Agarwal P Barbanel S Behrens C Berges A Burky J. Non-clinical immunological comparison of a Next-Generation 24-valent pneumococcal conjugate vaccine (VAX-24) using site-specific carrier protein conjugation to the current standard of care (PCV13 and PPV23). Vaccine. (2021) 39:3197–206. doi: 10.1016/j.vaccine.2021.03.070 33965258

[103] Wassil J Sisti M Fairman J Davis M Fierro C Bennett S. Evaluating the safety, tolerability, and immunogenicity of a 24-valent pneumococcal conjugate vaccine (VAX-24) in healthy adults aged 18 to 64 years: a phase 1/2, double-masked, dose-finding, active-controlled, randomised clinical trial. Lancet Infect Dis. (2023) 24(3):308–18. doi: 10.1016/S1473-3099(23)00572-8 38061367

