# Supplementary material for: Analysis of immunogenicity and purification methods in conjugated polysaccharide vaccines: a new approach in fighting pathogenic bacteria
Source: Front Immunol. 2024 Nov 20;15:1483740. doi: 10.3389/fimmu.2024.1483740 (PMC11614811; doi:10.3389/fimmu.2024.1483740)
Supplement: Supplementary file 1 [file Table1.docx]

Supplementary Material

Table 1 Conjugated vaccines in development for bacteria with no market-available vaccines

| **Bacteria** | **Method of conjugation** | **Basis of vaccine development** | **Studies** |
| --- | --- | --- | --- |
| Group B *Streptococcus* | CRM_197_ conjugate vaccines  (GBS6) | Various pathogenic elements produced by GBS are associated with processes such as colonization, adherence, invasion, and evasion of the immune system, with the potential to be considered candidates for vaccination. Among GBS’s extensively researched pathogenic elements, is its unique CPS, containing high levels of sialic acid. This CPS hinders complement deposition and shields bacteria from opsonophagocytosis by immune cells, thus aiding in evading the host's immune defenses. Additionally, the CPS facilitates biofilm formation, impedes the binding of antimicrobial peptides and neutrophil extracellular traps (NET), and disrupts bacterial adherence to epithelium and mucus, which enhances GBS invasiveness. GBS expresses ten CPS types (Ia, Ib, II–IX) with varying structures and antigenic properties. Comprising different arrangements of monosaccharides, the CPS includes a sialic acid residue at the terminal end of the repeating unit. Recent meta-analyses reveal that five prevalent GBS serotypes (Ia, Ib, II, III, and V) are accountable for 97% of invasive isolates globally, with serotype III being most commonly associated with infant diseases. Serotype IV is emerging as a significant cause of invasive diseases, particularly in non-pregnant adults, and shows the capability to become a critical factor in neonatal diseases, with some cases already documented. As polysaccharides (PSs) are antigens that do not necessitate T-cell activation, they are conjugated to a protein carrier to induce both protective and memory B-cell responses (1). | Previous vaccines have been linked to TT, a feature that could be particularly advantageous in regions with low and middle-income where neonatal tetanus is still a concern. Nevertheless, the primary carrier protein currently in use is CRM197, a harmless version of diphtheria toxin capable of inducing an immune response. Research using either carrier protein has shown that CPS-conjugates result in better immunogenicity with elevated levels of antibodies in contrast to vaccines that are not conjugated.  It has been proven that vaccines with multiple components are more efficient in generating immunity that can respond across various serotypes of GBS. Trials have indicated that a trivalent CRM197 conjugate vaccine given to pregnant women led to increased levels of CPS-specific antibodies in newborns. Additional clinical trials are required to confirm the safety and effectiveness of a hexavalent vaccine that targets multiple GBS serotypes (1).  A study on a novel hexavalent vaccine (GBS6) funded by Pfizer showed that overall, GBS6 showed good safety and tolerability at all doses and formulations, with only mild and short-lasting injection-site pain. Reactogenicity did not increase with higher doses. The vaccine had similar local and systemic reactions as other licensed vaccines for adults and investigational GBS vaccines for pregnancy. GBS6 induced strong immune responses, with rapid and sustained increases in antibody concentration up to 1 month after vaccination. While a threshold for protection has not been confirmed, An IgG concentration of no less than 1 μg/mL at the one-month mark was utilized as an initial evaluation of the immune response. Further studies are needed to determine protective thresholds for different serotypes and multivalent GBS vaccines (2). |
|  | Bioconjugate vaccine |  | A study was conducted on the development of a trivalent bioconjugate vaccine targeting the Ia, Ib, and III GBS serotypes. By utilizing the PglS system, fully sialylated bioconjugates of the three CPS serotypes were created. These bioconjugates were attached to a genetically inactivated form of *Pseudomonas aeruginosa* (*P. aeruginosa*) exotoxin A (EPA) containing a significant portion of the ComP protein, the natural substrate of PglS. The structural composition of each vaccine component was confirmed using various analytical methods, such as NMR spectroscopy on glycoengineered GBS PSs and mass spectrometry on intact bioconjugates.  The trivalent GBS vaccine tested in the study demonstrated the capability to evoke notable increases in serotype-specific IgG responses to all three capsule types. However, the response to the type III antigen was relatively weak. Additionally, it was shown that the antibodies produced by the GBS bioconjugate vaccine were functional, as evidenced by the opsonophagocytosis killing assay, a standard measure of immunity commonly used to evaluate the efficacy of *pneumococcal* conjugate vaccines. This assay is also frequently employed to assess the functional activity of vaccine-induced antibodies generated by GBS conjugate vaccines in clinical trials. Notably, the opsonophagocytic killing activity was most effective against type Ib and Ia GBS strains, reflecting the higher IgG titers for these types. In contrast, a modest opsonophagocytic killing response was seen in mice sera vaccinated with the trivalent GBS bioconjugate against the GBSIII strain. Nonetheless, the bioconjugate vaccine showed promise in generating functional antibody responses, as indicated by the opsonophagocytic killing assay. It is possible that enhancing the dose of the type III GBS component could further improve these responses, a strategy previously utilized with *pneumococcal* conjugate vaccines to induce comparable functional antibody responses for specific serotypes by doubling the concentration of the respective *pneumococcal* PS conjugates (3). |
| *Klebsiella* | hexasaccharide linked to the CRM197 carrier protein | various methods have been employed to create conjugate vaccines. One approach involves linking a hexasaccharide to a carrier protein, resulting in high IgG titers. Another study synthesized three OSs and conjugated them to a toxoid. Outer core tetra- and pentasaccharides were also synthesized and linked to carrier proteins. Alternatively, a PS can be purified and chemically conjugated to a carrier protein. Capsule depolymerases have been used to cleave capsules into OSs, which are then conjugated to a carrier protein. An alternative approach is to focus on O-antigen PSs develop conjugate vaccines. Bioconjugation, a specialized technique, leverages bacterial protein glycosylation systems to produce conjugate vaccines enzymatically. This strategy presents benefits compared to traditional chemical conjugation. The initial bioconjugate vaccines for *K. pneumoniae* effectively protected against severe pneumonia. Utilizing bioconjugation to link O2 O-antigen with cholera toxin B subunit led to the production of high levels of antibodies and offered protection against bloodstream infection. A comparison of bioconjugate vaccines targeting the K2 capsule or O1 O-antigen indicated that the capsule impedes the effectiveness of O-directed antibodies (4). | The synthesis of the CR-Kp CPS hexasaccharide RU 1 and its associated glycans 2–6 has enabled the examination of glycan microarrays. These investigations have indicated that hexasaccharide 1 is specifically recognized by the monoclonal antibody 1C9, which also exhibits cross-reactivity with CR-Kp CPS. The conjugate CRM197-1 has been proven to trigger elevated levels of antibody generation in both mice and rabbits, and it has displayed immunological memory in mice through its cross-reactivity with native CPS. Vaccination with the CRM197-1 conjugate has led to the development of CR-Kp CPS-specific antibodies that can attach to the surface of intact bacteria. This attachment has subsequently resulted in the in vitro phagocytic killing of CR-Kp *Klebsiella* strains. Nevertheless, a thorough assessment of CRM197-1's suitability as a vaccine candidate can only be carried out once an appropriate animal challenge model, currently being established for CR-Kp strains, becomes accessible (5). |
|  | K2 CPSs linked to DT |  | The initial chemical syntheses of the K2 CPS of KP were documented, resulting in the production of various lengths of chains. All desired molecules were furnished with an aminopentyl linker for protein conjugation. A highly selective method for the production of the desired molecules was established through the utilization of glycosyl fluorides and a tetrasaccharide acceptor. The produced glycans were identified through the use of NMR and mass spectrometry. Glycoconjugate vaccine candidates were developed by linking the synthesized glycans to carrier protein DT. Mice were vaccinated with these candidates, and their immunological response and antigenicity were assessed. Out of the three glycoconjugates, two were observed to be immunogenic, generating antibodies that recognized all synthetic glycans. The DT-Hepta 4aa glycoconjugate elicited a significant antibody response and displayed effective bactericidal activity. Heptasaccharide 4 emerges as a promising vaccine candidate for forthcoming animal trials. These investigations offer a framework for creating a vaccine targeting the K2 sero group of KP (6). |
|  | LPS PSs linked to CRM197 |  | The synthesis of pentasaccharide 1 and tetrasaccharide 2 from the outer core of *K. pneumoniae* LPS involved the use of monosaccharide building blocks with suitable amino- and hydroxyl-protecting groups. These outer core OSs are unique due to the presence of a Kdo and l-α-d-heptoside residue, which are typically seen in the inner core section of LPS. By utilizing TFA, Cbz, and azide as amino protecting groups, an acetamide and free amine could be installed, enabling selective conjugation of the OSs to carrier proteins. The application of Nap and Lev as orthogonal hydroxyl protecting groups facilitated the introduction of a crowded 4,5-branched Kdo moiety through sequential glycosylations in the correct sequence. These protecting groups were also used for subsequent acceptor synthesis. Thiol-maleimide coupling chemistry was utilized to link the OSs to BSA and CRM197. Immunization studies in mice demonstrated that the conjugates could stimulate LPS-specific antibody responses. Moreover, these antibodies could identify clinically relevant intact bacteria, suggesting the possibility of possessing neutralizing properties (7). |
|  | O-antigen serotypes linked to flagellin proteins from *P. aeruginosa* |  | KP OPS was isolated from fermentation cultures of KP reagent strains and purified through a scalable manufacturing method to produce abundant quantities of highly pure PS with minimal residual protein and endotoxin. Analysis using HPLC-SEC revealed that the purified PSs were relatively uniform in size and consistent across the four OPS varieties. Monosaccharide composition assessments confirmed that KP O1 and O2 consisted of polygalactans, while KP O3 and O5 were composed of mannose. Purified PA FlaA1 and FlaB, produced as recombinant proteins in *Escherichia coli* (*E. coli*), exhibited protein bands at the expected molecular weight and distinct peaks in HPLC-SEC. The KP OPS molecules were conjugated to PA Fla protein lysines using a specific linker, forming neoglycoconjugates with intact OPS chains. Analysis with HPLC-SEC of the purified glycoconjugates suggested that each maintained a narrow size distribution and approximately equal PS-to-protein ratios. Ultimately, it was observed that linking rFlaA and rFlaB abolished TLR5 signaling. PA flagellin is an efficient carrier for KP OPS, with rFlaA and rFlaB performing equally as carriers. Antibodies against flagellin generated post-immunization with the conjugate vaccine guard against burn wound infection. However, antibodies against rFlaA offer limited protection and fail to hinder motility with PAK. The lengthier carbohydrate modifications on FlaA might hinder antibody binding to the protein surface. Recombinant FlaA could be misfolded, impacting protective epitopes. Alternatively, PAK might suppress flagellin in vivo due to alginate production (8). |
|  | K1 and K2 bioconjugate |  | Developing a K1 and K2 bioconjugate vaccine in *E. coli* requires the utilization of the oligosaccharyltransferase (OTase) PglS to attach PSs to a genetically modified form of exotoxin A protein. This protein is linked to a section of ComP, the natural receptor protein of PglS. The glycoproteins were isolated and analyzed using chromatography and staining techniques. The bioconjugate vaccines displayed atypical smear-like patterns, a common occurrence in glycoconjugate vaccines produced through chemical means. Typically, only three elements are needed for the effective attachment of PSs to proteins: an OTase (enzyme for conjugation), a target protein for glycosylation, and a PS for transfer. However, our research suggests that a fourth factor, the RmpA transcriptional activator, is also essential for the efficient production of K1 and K2 capsules in *E. coli*, necessitating further examination to understand the impact of different rmpA alleles on PS expression. The K1-EPA and K2-EPA bioconjugates were evaluated for their capacity to stimulate serotype-specific IgG responses in mice. Four groups of immunized mice were administered a placebo, the K1-EPA bioconjugate, the K2-EPA bioconjugate, or a combination of the K1- and K2-EPA bioconjugates. All vaccines were combined with Imject Alum as an adjuvant. The mice received three vaccinations, and serum samples were collected periodically to analyze the IgG responses. Mice immunized with K1-EPA exhibited elevated K1-specific IgG levels, and the bivalent vaccine also resulted in increased K1-specific IgG levels. No cross-reactivity was detected in mice vaccinated with K2-EPA. Mice immunized with K2-EPA displayed heightened K2-specific IgG levels, except for one mouse. The bivalent vaccine also led to increased K2-specific IgG levels. The kinetics of the IgG responses demonstrated that mice vaccinated with K1-EPA had a more pronounced response than those receiving the bivalent vaccine. A similar trend was observed in the case of K2-specific IgG responses. Analysis of the pooled sera from vaccinated mice revealed an exclusive IgG1-specific response to the corresponding antigens (9). |
| *Shigella* | Bioconjugate | Despite thorough investigation and the extensive array of vaccine candidates that have been subjected to clinical trials in an endeavor to create a safe and efficient *Shigella* vaccine with broad serotype coverage, an easily accessible licensed vaccine remains elusive. Despite some limitations, the surface polysaccharides of *Shigella*, particularly the O-antigens, persist as crucial targets for vaccine development. Progressing beyond initial accomplishments in *Shigella* lipopolysaccharide (LPS)-based conjugate vaccines, novel concepts that integrate glycoscience and molecular vaccinology offer promising avenues for creating more precise and immunogenic *Shigella* glycoconjugate vaccines. The rapidly evolving in vivo glycoengineering technology introduces a fresh and possibly cost-effective approach to vaccine development against a significant global health pathogen. Similarly, by harnessing the latest advancements in chemical and structural biology, the synthetic glycan-based strategy presents a distinctive chance to enhance our comprehension of immune parameters applicable to glycoconjugate vaccine-induced protection. This knowledge can be utilized to devise enhanced *Shigella* carbohydrate antigens and tailored, efficient conjugate vaccines that imitate the natural diverse polysaccharides. Moreover, acknowledging the essential role of the carbohydrate component in molding the adaptive immune response to glycoconjugates opens up new pathways for creating highly protective knowledge-based carbohydrate-based vaccines. While concerns related to manufacturing have impeded early progress, the synthetic glycan-based strategy is becoming increasingly appealing due to its demonstrated feasibility on an industrial scale and the continual diversification of methodologies, including chemoenzymatic approaches, one-pot protocols, automated solid-phase synthesis, and advancements in conjugation chemistry. In recent years, monovalent *Shigella* vaccine candidates embodying the swiftly expanding bioconjugate and synthetic glycan-based strategies have successfully traversed several stages of clinical trials in volunteers from Western nations. Despite potential concerns regarding novelty, the current advancement of strategies akin to those of the "sun" type offers distinct advantages compared to the original approaches in terms of production, characterization, integrity, control, and versatility of the product to achieve efficacy. Nevertheless, lacking an appropriate animal model and a clear consensus on the critical protective factors, even though emerging research proposes that various components of the immune system contribute to immunity against shigellosis, future progress will heavily hinge on two factors: (i) showing the viability of vaccine candidates capable of provoking an immune response against a variety of strains responsible for the disease, considering the wide range of antigens, and (ii) conducting clinical trials to ascertain the immunogenicity of the vaccine in the designated populations and gain insights into its potency. In a landscape where the burden of *Shigella* is garnering renewed attention, heightened efforts and resources from new funding partners have paved the way for swift advancements toward these objectives while also facilitating collaborative contributions from a diverse array of experts committed to combatting the burden of *Shigella* (10). | The phase 2b challenge trial has provided clinical evidence of the effectiveness of the monovalent bioconjugate vaccine in combatting severe shigellosis outcomes. Subsequent trials will assess the potential of the quadrivalent bioconjugate vaccine in eliciting strong immunogenicity and effectively fighting the disease. The safety profile of the vaccine was deemed satisfactory, similar to the observations made in the initial phase of the study. Regarding immunogenicity, following the initial dose, there was a tenfold increase in IgG responses compared to the baseline. Additional doses or subsequent challenges did not result in further elevation of antibody levels in vaccine recipients, which remained notably higher than those in the placebo group. The investigation also delved into other factors like memory B-cell responses and gut-homing LPS-specific antibody responses, all of which supported the vaccine's ability to trigger a robust immune reaction. The effectiveness of the bioconjugate was verified through various analyses, including a shigellosis disease score, which indicated a reduction in disease severity among vaccinated individuals compared to those who received a placebo, suggesting that vaccination could mitigate the severity of the disease even if it does not entirely prevent it. Moreover, the study highlighted those immune responses triggered by vaccination, particularly the presence of 2a-LPS specific serum IgG, were strongly linked to protection against shigellosis post-oral challenge, with higher levels of anti-Sf2a LPS IgG correlating with decreased bacterial replication in the gut. Additionally, the level of anti-SF2a LPS-serum IgG remained notably higher in the vaccinated group even one year after vaccination, indicating a sustained immune response. The study also discovered that parenteral immunization stimulated mucosal immunity and that mucosal responses were connected to defense against the disease (11). |
|  | Synthetic Glycan-Based Conjugate |  | The article delved into the GMP manufacturing process of SF2a-TT15, a vaccine contender for shigellosis. The production capacity was successfully raised to 100 mL, showcasing the strength of the procedure. The utilization of TFF in the process was deemed viable. The final production resulted in thousands of doses of SF2a-TT15 that adhered to all GMP standards and displayed potent immunogenic properties. The enduring stability of SF2a-TT15 positions it as a hopeful candidate for thiol-maleimide bioconjugation in advancing glycoconjugate vaccines. The initial examination indicated the vaccine's safety and efficacy in triggering elevated antibodies against SF2a bacteria. Subsequent research in mice and humans validated the promise of SF2a-TT15 as a vaccine prospect. Future research will focus on evaluating its protective capacity in humans and assessing its safety and immunogenicity in specific populations. This report highlights the feasibility and importance of developing synthetic carbohydrate-protein conjugate vaccines for infectious diseases (12). |
|  | Glyconjugate |  | The utilization of glycoconjugate methodology in the development of *Shigella* vaccines has been inspired by the successful application of this approach in creating vaccines against various other bacterial strains. *Shigella* bacteria are distinctive in that they do not possess capsules, which is why the O-antigen of Lipopolysaccharides (LPS) is utilized in these vaccines. The immune response elicited by the O-antigen plays a crucial role in safeguarding against shigellosis. Extensive research has been conducted on developing glycoconjugate vaccines, showing encouraging results in clinical trials. Notably, the *Shigella* *sonnei* (*S. sonnei*) O-antigen/rEPA vaccine exhibited remarkable efficacy, although a glycoconjugate vaccine has not yet been officially approved for use. Protection has been demonstrated in military personnel, yet the primary target demographic for a universal *Shigella* vaccine consists of young children in low- and middle-income countries (LMICs). A subsequent efficacy study displayed protection against *S. sonnei* shigellosis in children aged 3-4 years but not in those under three years old. The decline in serum O-antigen IgG levels correlated with losing protective effects. Despite these advancements, the commercialization of such vaccines has not yet transpired (13).  The experimental vaccine ZF0901, produced by Beijing Zhifei Lvzhu Biopharmaceutical Co., Ltd. in China, is a conjugate vaccine comprising O-SP extracted from *Shigella* *flexneri* (*S. flexneri*) 2a or *S. sonnei*, linked to TT via adipic acid dihydrazide. It complies with standards for endotoxin content and injection volume. A complete dose contains 10 µg of *S. Sonnei* O-SP, 10 µg of *S. Flexneri* 2a O-SP, and 50 µg of TT, while a half-dose includes 5 µg of *S. Sonnei* O-SP, 5 µg of *S. Flexneri* 2a O-SP, and 25 µg of TT.  The research demonstrated the safety and efficacy of the ZF0901 vaccine in neonates and young children. No severe adverse reactions were observed, with fever being the most frequent side effect. Incidence of fever was consistent across all groups, and rates of diarrhea, nausea/vomiting, and dysphoria/irritability did not significantly differ between vaccine and control groups. Rates of unsolicited adverse drug reactions were comparable between ZF0901 vaccine recipients and the control group.  The ZF0901 vaccine prompted a notable rise in type-specific IgG antibodies against *S. flexneri* 2a and *S. sonnei* in all vaccine recipients. Over half of the subjects experienced a significant increase in antibody levels regardless of dosage or number of injections. There were hints that the impact of alum adjuvant varied among age groups, but these variances were not statistically significant (14). |

1. Carreras-Abad C, Ramkhelawon L, Heath PT, Le Doare K. A Vaccine Against Group B Streptococcus: Recent Advances. Infection and drug resistance. 2020;13(null):1263-72.

2. Absalon J, Segall N, Block SL, Center KJ, Scully IL, Giardina PC, et al. Safety and immunogenicity of a novel hexavalent group B streptococcus conjugate vaccine in healthy, non-pregnant adults: a phase 1/2, randomised, placebo-controlled, observer-blinded, dose-escalation trial. The Lancet Infectious Diseases. 2021;21(2):263-74.

3. Duke JA, Paschall AV, Robinson LS, Knoot CJ, Vinogradov E, Scott NE, et al. Development and Immunogenicity of a Prototype Multivalent Group B Streptococcus Bioconjugate Vaccine. ACS Infectious Diseases. 2021;7(11):3111-23.

4. Wantuch PL, Rosen DA. Klebsiella pneumoniae: adaptive immune landscapes and vaccine horizons. Trends in Immunology. 2023.

5. Seeberger PH, Pereira CL, Khan N, Xiao G, Diago-Navarro E, Reppe K, et al. A Semi-Synthetic Glycoconjugate Vaccine Candidate for Carbapenem-Resistant Klebsiella pneumoniae. Angewandte Chemie International Edition. 2017;56(45):13973-8.

6. Ravinder M, Liao K-S, Cheng Y-Y, Pawar S, Lin T-L, Wang J-T, et al. A Synthetic Carbohydrate–Protein Conjugate Vaccine Candidate against Klebsiella pneumoniae Serotype K2. The Journal of Organic Chemistry. 2020;85(24):15964-97.

7. Chen D, Srivastava AK, Dubrochowska J, Liu L, Li T, Hoffmann JP, et al. A Bioactive Synthetic Outer-Core Oligosaccharide Derived from a Klebsiella pneumonia Lipopolysaccharide for Bacteria Recognition. Chemistry (Weinheim an der Bergstrasse, Germany). 2023;29(25):e202203408.

8. Hegerle N, Choi M, Sinclair J, Amin MN, Ollivault-Shiflett M, Curtis B, et al. Development of a broad spectrum glycoconjugate vaccine to prevent wound and disseminated infections with Klebsiella pneumoniae and Pseudomonas aeruginosa. PLOS ONE. 2018;13(9):e0203143.

9. Feldman MF, Mayer Bridwell AE, Scott NE, Vinogradov E, McKee SR, Chavez SM, et al. A promising bioconjugate vaccine against hypervirulent <i>Klebsiella pneumoniae</i>. Proceedings of the National Academy of Sciences. 2019;116(37):18655-63.

10. Barel L-A, Mulard LA. Classical and novel strategies to develop a Shigella glycoconjugate vaccine: from concept to efficacy in human. Human Vaccines & Immunotherapeutics. 2019;15(6):1338-56.

11. Martin P, Alaimo C. The Ongoing Journey of a Shigella Bioconjugate Vaccine. Vaccines. 2022;10(2):212.

12. van der Put RMF, Smitsman C, de Haan A, Hamzink M, Timmermans H, Uittenbogaard J, et al. The First-in-Human Synthetic Glycan-Based Conjugate Vaccine Candidate against Shigella. ACS Central Science. 2022;8(4):449-60.

13. MacLennan CA, Grow S, Ma L-f, Steele AD. The Shigella Vaccines Pipeline. Vaccines. 2022;10(9):1376.

14. Mo Y, Fang W, Li H, Chen J, Hu X, Wang B, et al. Safety and Immunogenicity of a Shigella Bivalent Conjugate Vaccine (ZF0901) in 3-Month- to 5-Year-Old Children in China. Vaccines. 2022;10(1):33.
